# UBE2T‐Driven p53 Degradation Rewires Glycolysis to Orchestrate Lactylation‐Mediated CAFs Activation and ECM Deposition in Pancreatic Cancer

**DOI:** 10.1002/advs.202514933

**Published:** 2026-02-15

**Authors:** Yong Ma, Wenbo Liu, Mingdou Li, Tao Wang, Bin Zhao, Keshen Wang, Qichen He, Haonan Sun, Huiguo Qing, Xiaoying Guan, Wengui Shi, Long Qin, Yuman Dong, Huinian Zhou, Zeyuan Yu, Xiangyan Jiang, Zuoyi Jiao

**Affiliations:** ^1^ The Second Hospital & Clinical Medical School Lanzhou University Lanzhou China; ^2^ Department of General Surgery Lanzhou University Second Hospital Lanzhou China; ^3^ Gansu Tumor Immunology Basic Disciplines Research Center The Second Hospital & Clinical Medical School Lanzhou University Lanzhou China; ^4^ Department of Neurosurgery Lanzhou University Second Hospital Lanzhou China; ^5^ Department of Pathology Lanzhou University Second Hospital Lanzhou China; ^6^ Cuiying Biomedical Research Center Lanzhou University Second Hospital Lanzhou China

**Keywords:** immunotherapy, lactylation, pancreatic cancer, tumor microenvironment, UBE2T

## Abstract

Although glycolysis is a metabolic hallmark of pancreatic ductal adenocarcinoma (PDAC), it remains unclear whether the excessive lactate produced regulates CAF lactylation to promote extracellular matrix (ECM) deposition. The multi‐omics and spontaneous model findings indicate that lactate accumulation in the tumor microenvironment (TME) promotes histone H3 lysine 18 lactylation (H3K18la) and activation of cancer‐associated fibroblasts (CAFs), leading to both ECM densification and impaired immunotherapy efficacy in PDAC. Mechanistically, ubiquitin‐conjugating enzyme E2T (UBE2T) acts as an initiating factor that promotes p53 positive feedback degradation through modulation of ribosome biogenesis, thereby enhancing lactate metabolic crosstalk via glycolytic reprogramming. Genetic ablation or pharmacological inhibition of UBE2T using the selective inhibitor pentagalloylglucose (PGG) disrupts lactate metabolic crosstalk, suppresses stromal deposition, and promotes intratumoral CD8^+^ T cells infiltration. Furthermore, the combination of PGG and anti‐PD‐1 therapy exhibits synergistic effects and survival benefits in spontaneous PDAC mice and immune‐reconstituted patient‐derived xenografts. Collectively, these findings reveal that UBE2T drives p53 positive feedback degradation to enhance glycolysis of PDAC, leading to excessive lactate production, which promotes H3K18la in CAFs and subsequent ECM deposition. Targeting UBE2T represents a potential strategy to improve the efficacy of immunotherapy in PDAC.

## Graphical abstract

This study integrates multi‐omics to reveal the critical role of UBE2T in driving malignancy and stromal co‐evolution in PDAC. UBE2T potentiates glycolysis by regulating p53 degradation via a positive feedback loop, thereby promoting histone H3 lysine 18 lactylation in CAFs and stromal deposition. The UBE2T inhibitor PGG represents a potential strategy to improve the efficacy of immunotherapy in PDAC.

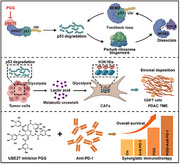

## 1 Introduction

Pancreatic ductal adenocarcinoma (PDAC) remains one of the most lethal malignancies, with a dismal 5‐year survival rate of 13% [1]. While immune checkpoint blockade therapies targeting programmed death‐1 (PD‐1) have revolutionized cancer treatment, PDAC exhibits remarkable therapeutic resistance except the rare with microsatellite instability [2, 3]. This therapeutic recalcitrance is largely attributed to its unique tumor microenvironment (TME), characterized by a densely fibrotic stroma constituting up to 90% of tumor volume [4, 5]. The dense extracellular matrix (ECM) is primarily generated by cancer‐associated fibroblasts (CAFs), forming a physical barrier that impedes drug penetration and immune cell infiltration. Concurrently, CAFs contribute to the establishment of an immunosuppressive microenvironment through cytokine secretion and ECM remodeling [6, 7]. The clinical failures of stroma‐modulating agents underscore the intricate complexity of stroma‐tumor crosstalk [8, 9]. In this context, elucidating the mechanisms driving stromal deposition in PDAC is critical for developing strategies to improve immunotherapy efficacy.

Metabolic reprogramming is a hallmark of PDAC, not only providing energy for tumor cell survival and function maintenance but also contributing to TME remodeling [10, 11]. Aerobic glycolysis serves as the predominant energy metabolism mode in cancer cells [12]. Under the influence of tumor cells, CAFs undergo a significant metabolic shift from oxidative phosphorylation to aerobic glycolysis, a phenomenon termed the reverse “Warburg effect” [13]. These metabolic alterations collectively promote the formation of a hypoxic, acidic TME that supports tumor growth and therapeutic resistance. Lactate, a byproduct of glycolysis, facilitates cancer cell secreted soluble cytokines to mediate CAF‐tumor cell communication, promoting ECM remodeling and immune suppression [7, 14]. CAFs can also utilize lactate produced by enhanced glycolysis in cancer cells as an energy source to maintain a dense, immunosuppressive TME [15]. Recently, studies have revealed that lactate accumulation in the TME induces a novel post‐translational modification (PTM) termed lactylation, which is closely associated with tumor malignant progression, immune suppression and therapy resistance [16, 17, 18, 19]. Furthermore, histone H3 lysine 18 lactylation (H3K18la) promotes fibroblast activation, thereby inducing tissue fibrosis [20]. However, the role of lactylation in the dense stromal microenvironment of PDAC remains unclear.

In this study, we demonstrated that lactate‐mediated histone lactylation of CAFs is closely associated with stromal deposition in PDAC. Mechanistically, UBE2T drives p53 positive feedback degradation through regulating ribosome biogenesis, thereby enhancing glycolysis and excessive lactate production. This subsequently upregulates H3K18la levels in CAFs, promoting ECM densification. Importantly, we further showed that targeting UBE2T suppresses stromal deposition and synergizes with PD‐1 blockade, offering a promising combination therapy strategy to improve the effectiveness of immunotherapy for PDAC.

## 2 Results

### 2.1 Lactate‐Mediated H3K18 Lactylation in CAFs Drives CAF Activation and Stromal Deposition in PDAC

**FIGURE 1:**
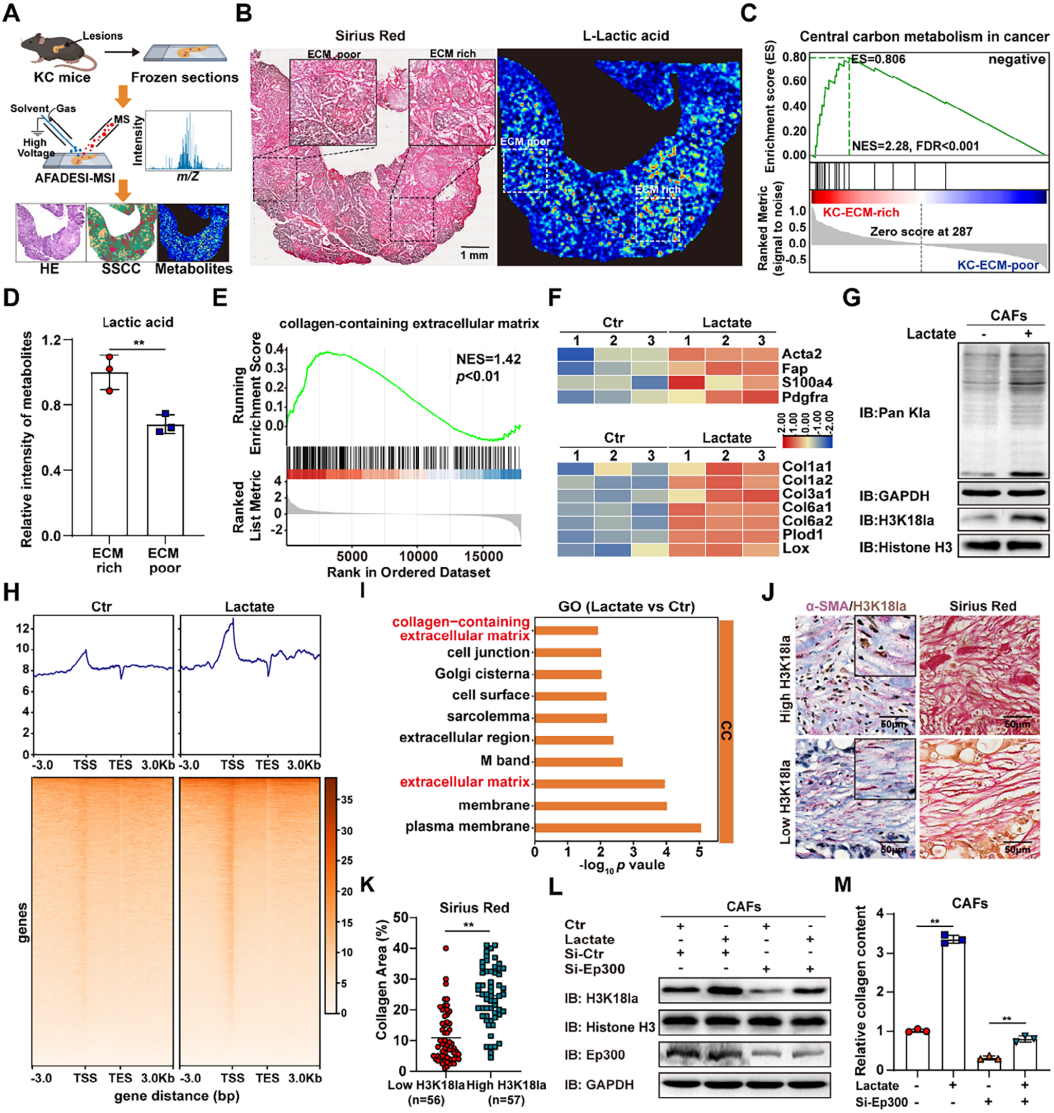
Lactate promotes H3K18la in CAFs and stromal densification. (A) Schematic diagram of spatial metabolomics analysis. (B) Sirius Red staining and mass spectrometry imaging (MSI) of lactate in pancreatic tissues of KC mice. (C) Gene Set Enrichment Analysis (GSEA) of central carbon metabolism in ECM‐rich and ECM‐poor regions based on spatial metabolomics data. (D) Lactate levels in ECM‐rich and ECM‐poor regions based on spatial metabolomics analysis (*n* = 3). (E) Gene Ontology (GO) analysis of collagen‐containing extracellular matrix in CAFs with or without lactate treatment (10 mm). (F) Heatmap illustrating expression levels of activation‐related genes (top) and ECM‐related genes (bottom) in CAFs with or without lactate treatment (*n* = 3). (G) Protein levels of Pan Kla and H3K18la in CAFs with or without lactate treatment (10 mm). (H) Heatmaps for H3K18la binding peaks in CAFs with or without lactate treatment. (I) GO analysis of genes associated with H3K18la binding peaks in CAFs with or without lactate treatment. (J,K) Representative images of H3K18la/α‐SMA and Sirius Red staining(J), and quantification of Sirius Red staining with low or high H3K18la expression in CAFs(K). (L) Protein levels of H3K18la in Si‐Ctr and Si‐Ep300 CAFs with or without lactate treatment (10 mm). (M) Relative collagen content in Si‐Ctr and Si‐Ep300 CAFs with or without lactate treatment (*n* = 3). Student's *t* test in D, K and M, results are presented as the mean ± SD. ^**^ *p* < 0.01.

To investigate the relationship between lactate accumulation and stromal deposition in the PDAC TME, we generated a spontaneous PDAC model harboring *LSL‐Kras^G12D/+^* and *Pdx1‐Cre* (KC), followed by spatial metabolomic analysis of pancreatic tissues (Figure 1A). Sirius Red staining assessed ECM deposition levels, and differential metabolite analysis between ECM‐rich and ECM‐poor regions revealed enhanced central carbon metabolism and higher lactate accumulation in ECM‐rich region (Figure 1B–D; Figure S1A–C). To elucidate the effect of lactate on ECM secretion by CAFs, we isolated CAFs from a spontaneous PDAC model harboring *LSL‐Kras^G12D/+^*, *LSL‐p53^R172H/+^*, and *Pdx1‐Cre* (KPC). Our data demonstrated that exogenous lactate treatment promoted collagen secretion by CAFs (Figure S1D). Transcriptomic analysis showed that lactate‐treated CAFs exhibited ECM‐like alterations, with upregulated expression of CAF activation markers and collagen synthesis genes compared to controls (Figure 1E,F; Figure S1E). Given the role of lactylation in pathological fibrosis [20], we evaluated the effects of lactate on pan‐lactylation (Pan Kla) and histone lactylation levels in CAFs. The results showed that lactate significantly enhanced global lactylation levels, with H3K18 among histones showing the most prominent change (Figure 1G; Figure S1F). Next, we performed CUT&Tag analysis using anti‐H3K18la antibody to delineate the regulatory role of H3K18la in lactate‐treated CAFs. The analysis revealed H3K18la was significantly enriched at transcription start site (TSS) regions in the lactate‐treated group, with a substantial number of H3K18la binding peaks located within promoter sequences (Figure 1H). Combined with transcriptomic analysis, it was found that H3K18la enrichment was increased at the promoters of Acta2 and Col1a1 in lactate‐treated CAFs (Figure S1H). Gene Ontology (GO) analysis of genes associated with H3K18la enrichment also indicated their involvement in the regulation of the extracellular matrix (Figure 1I; Figure S1G). Further analysis of PDAC clinical samples revealed a significant positive correlation between H3K18la protein levels in CAFs and stromal deposition within the PDAC TME (Figure 1J,K; Figure S1I). Next, we knocked down *Ep300*, a potential writer of histone lactylation, in CAFs to demonstrate that lactate‐mediated H3K18la is a critical factor for stromal deposition. The results showed that *Ep300* knockdown attenuated the regulatory effects of lactate on H3K18la and collagen secretion (Figure 1L,M). Collectively, these findings suggest that lactate accumulation upregulates H3K18la levels in CAFs, thereby promoting stromal deposition in the PDAC TME.

### 2.2 Targeting Lactate Metabolism Attenuates Stromal Deposition and Reinvigorates Anti‐Tumor Immunity

**FIGURE 2:**
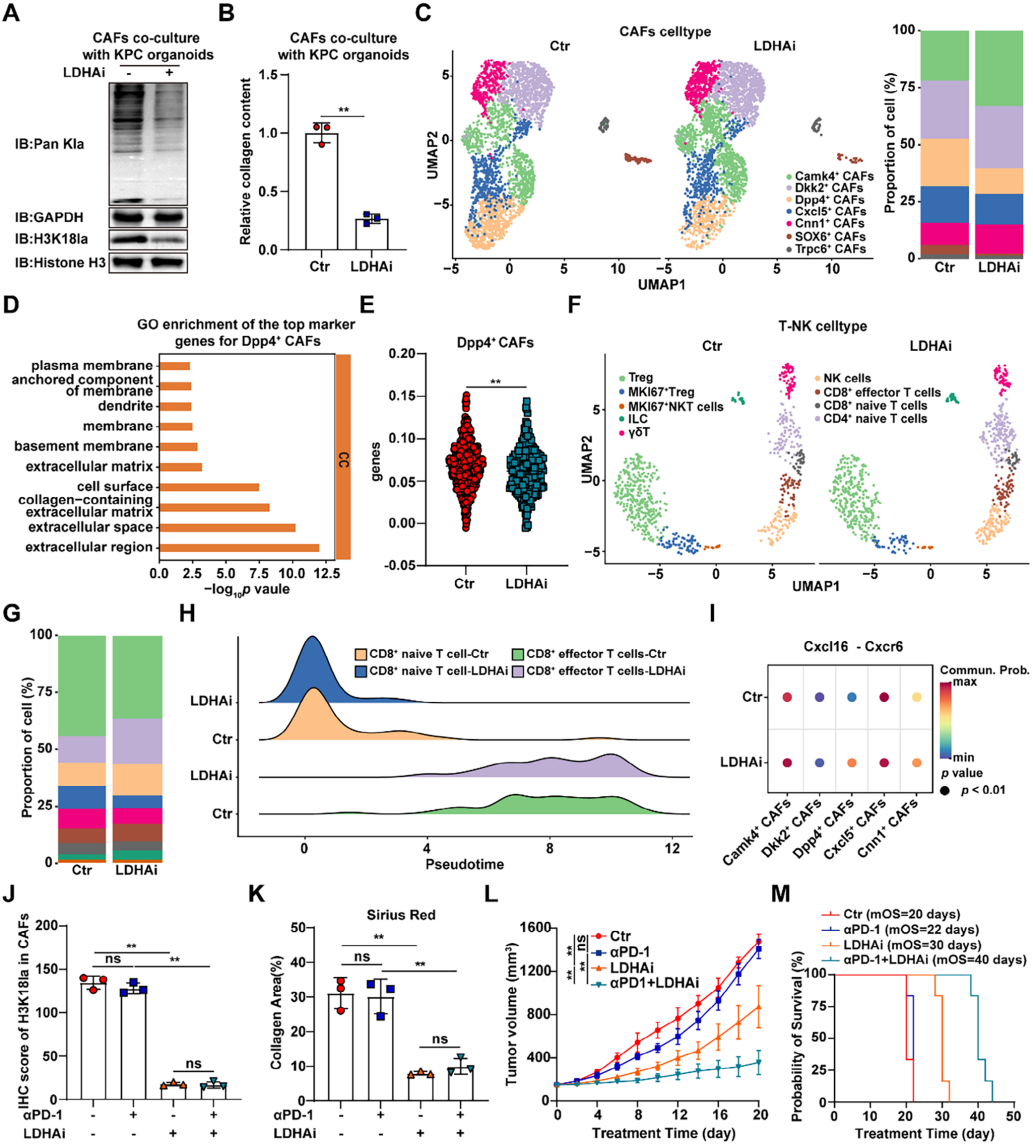
Inhibition of LDHA reduces stromal deposition and enhances immunotherapy efficacy in PDAC. (A) Protein levels of Pan Kla and H3K18la in CAFs co‐cultured with KPC organoids treated with or without oxamate (10 mm). (B) Relative collagen content in CAFs co‐cultured with KPC organoids treated with or without oxamate (*n* = 3). (C) Uniform manifold approximation and projection (UMAP) plot showing scRNA‐seq annotations and proportions of CAFs cell type in KPC allografts treated with or without oxamate. (D) GO analysis of Dpp4^+^ CAFs based on top 100 marker genes. (E) Immunosuppression score in Dpp4^+^ CAFs from KPC allografts with or without oxamate analyzed by scRNA‐seq. (F,G) UMAP plot showing scRNA‐seq annotations (F) and proportions (G) of T‐NK cell type in KPC allografts treated with or without oxamate. (H) Ridge plot of CD8^+^ T cell subtype proportions across pseudotime from KPC allografts with or without oxamate analyzed by scRNA‐seq. (I) Bubble plot showing the interaction strength of the Cxcl16‐Cxcr6 axis between CAF subsets and effector CD8^+^ T cells from KPC allografts with or without oxamate analyzed by scRNA‐seq. (J,K) Quantification of H3K18la/α‐SMA (J), Sirius Red staining (K) in KPC allografts treated with or without oxamate and/or anti‐PD‐1 therapy (*n* = 3). (L,M) Tumor growth (L) and OS analysis (M) of KPC allografts treated with or without oxamate and/or anti‐PD‐1 therapy (*n* = 6). Student's *t* test in B and E, One‐way ANOVA with Bonferroni correction in J–L, results are presented as the mean ± SD. ^**^ *P* < 0.01; ns, no significance.

To investigate the effects of inhibiting lactate metabolism in tumor cells on CAFs, we co‐cultured KPC organoids pre‐treated with the LDHA inhibitor oxamate and CAFs, and found that H3K18la and collagen secretion levels in the co‐cultured CAFs were significantly decreased. (Figure 2A,B). To further elucidate the impact of lactate metabolism on the PDAC TME, we treated KPC allograft mice with oxamate and performed single‐cell RNA sequencing analysis (Figure S2A,B). We observed a significant reduction in Dpp4^+^ CAFs associated with extracellular matrix regulatory functions (Figure 2C,D; Figure S2C,D). Previous studies have reported that DPP4 can promote the transition of iCAFs to the ECM‐myCAF state and promote pathological tissue fibrosis [21, 22]. Upon co‐culturing *Ldha*‐knockdown organoids with CAFs, we observed decreased Dpp4 protein levels and reduced collagen secretion in CAFs (Figure S2E). Scoring of the negative regulation of immune response (GO:0050777) in Dpp4^+^ CAFs revealed a marked decrease in their immunosuppressive following oxamate intervention (Figure 2E). Analysis of T‐NK cells showed that LDHA inhibition led to a reduced proportion of immunosuppressive Treg cells and an increased proportion of immune effector cells, including NK cells and CD8^+^ effector T cells (Figure 2F,G; Figure S2F). Concurrently, the immunosuppressive activity of Tregs was diminished, while the expression of functional effector genes in CD8^+^ effector T cells was upregulated (Figure S2G,H). Pseudotime analysis indicated enhanced differentiation from naïve to effector phenotypes in CD8^+^ T cells after LDHA inhibition (Figure 2H; Figure S2I). Cell‐cell communication analysis revealed that after LDHA inhibition, the interaction between Dpp4^+^ CAFs and effector CD8^+^ T cells was significantly enhanced, particularly through the CXCL16‐CXCR6 ligand‐receptor pair (Figure 2I), which has been reported to promote CD8^+^ T cell tissue infiltration in pancreatic cancer [23]. Subsequently, we evaluated the impact of LDHA inhibition on anti‐PD‐1 therapy in KPC allograft model. Oxamate reduced H3K18la levels in CAFs and stromal deposition, while combination therapy with anti‐PD‐1 significantly enhanced CD8^+^ T cell infiltration (Figure 2J,K, Figure S3A–E). Moreover, oxamate and anti‐PD‐1 combination therapy suppressed tumor progression and prolonged survival in KPC mice (Figure 2L,M; Figure S3F–H).

### 2.3 Positive‐Feedback Degradation of p53 Driven by UBE2T Promotes CAF Lactylation and Stromal Deposition

**FIGURE 3:**
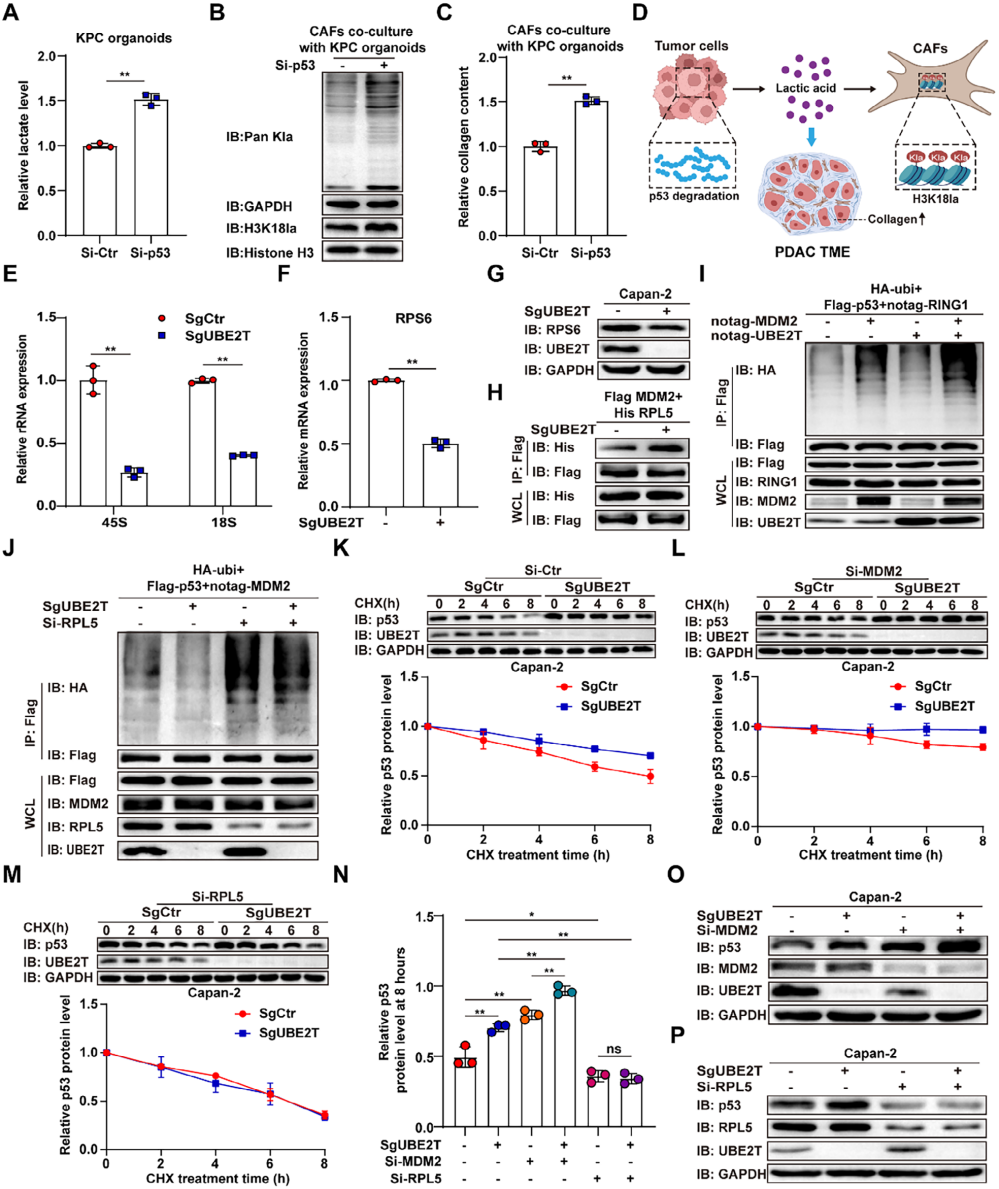
Positive‐feedback degradation of p53 mediated by UBE2T promotes CAF lactylation and stromal deposition. (A) Relative lactate levels in KPC organoids with or without *p53* knockdown (*n* = 3). (B) Protein levels of Pan Kla and H3K18la in CAFs co‐cultured with KPC organoids with or without *p53* knockdown. (C) Relative collagen content in CAFs co‐cultured with KPC organoids with or without *p53* knockdown (*n* = 3). (D) Schematic illustration of p53 degradation promoting CAF lactylation to enhance ECM deposition. (E) 45S and 18S rRNA expression levels in SgCtr and SgUBE2T Capan‐2 cells (*n* = 3). (F,G) RPS6 mRNA expression (*n* = 3) (F) and protein levels (G) in SgCtr and SgUBE2T Capan‐2 cells. (H) CoIP assays showing the interaction between MDM2 and RPL5 in SgCtr and SgUBE2T Capan‐2 cells. (I,J) Ubiquitination assay showing the degree of p53 ubiquitination in Capan‐2 cells expressing the indicated plasmids. (K–N) Immunoblotting assay shows the half‐life of p53 proteins treated with 100 ug mL^−1^ cycloheximide (CHX) (*n* = 3) in SgCtr and SgUBE2T Capan‐2 cells with or without *MDM2/RPL5* knockdown. (O,P) p53 protein levels in SgCtr or SgUBE2T Capan‐2 cells with or without *MDM2/RPL5* knockdown. Student's *t* test in A,C,E and F, One‐way ANOVA with Bonferroni correction in N, results are presented as the mean ± SD. ^*^ *p* < 0.05; ^**^ *p* < 0.01; ns, no significance.

Extensive studies have demonstrated that p53 regulates multiple metabolic enzymes to suppress glycolysis, and its loss in tumor cells fosters an immunosuppressive tumor microenvironment, thereby inducing immune tolerance [24, 25]. To investigate the role of p53 in lactate secretion and stromal deposition, we co‐cultured *p53*‐knockdown organoids with CAFs. Following *p53* knockdown, lactate accumulation in the culture medium increased, and co‐cultured CAFs exhibited elevated Pan Kla and H3K18la protein levels as well as increased collagen secretion (Figure 3A–C). These findings suggest that p53 degradation in tumor cells promotes lactate production, which in turn increases H3K18la levels in CAFs, leading to stromal deposition (Figure 3D).

Our previous research demonstrated that the ubiquitin‐conjugating enzyme UBE2T interacts with the E3 ligase RING1 to mediate K48‐linked ubiquitination and subsequent degradation of p53 (Figure S4A) [26]. Additionally, p53 suppresses ribosome biogenesis, and impaired ribosome biogenesis enhances ribosomal protein binding to MDM2, thereby inhibiting p53 ubiquitination and degradation [27, 28]. Building upon these observations, we propose that UBE2T‐mediated ubiquitination of p53 may operate through a feedback regulatory mechanism. We observed that *UBE2T* knockout reduced both mRNA and protein levels of ribosomal protein S6 (RPS6), along with decreased levels of 45S and 18S rRNAs, indicating impaired ribosome biogenesis (Figure 3E–G; Figure S4B). Co‐immunoprecipitation revealed that *UBE2T* depletion enhanced the binding affinity between ribosomal protein L5 (RPL5) and MDM2 (Figure 3H). Ubiquitination assays demonstrated that UBE2T potentiated MDM2‐mediated p53 ubiquitination, while RPL5 knockdown attenuated this effect (Figure 3I,J). In addition, excessive lactate can inhibit the promoting effect of UBE2T on MDM2‐mediated p53 ubiquitination (Figure S4C). Cycloheximide chase experiments confirmed that UBE2T promotes MDM2‐mediated p53 degradation in an RPL5‐dependent manner (Figure 3K–N; Figure S4D,E). Consistently, *MDM2* knockdown further elevated p53 protein levels in *UBE2T*‐depleted cells, whereas *RPL5* knockdown abolished the differential p53 protein levels between control and *UBE2T*‐knockout groups (Figure 3O,P). Collectively, these findings establish UBE2T as a priming factor that promotes MDM2‐mediated positive‐feedback degradation of p53 by modulating ribosome biogenesis (Figure S4F).

### 2.4 The UBE2T‐p53 Axis Drives Lactate Metabolic Crosstalk and Stromal Deposition via Glycolytic Rewiring

**FIGURE 4:**
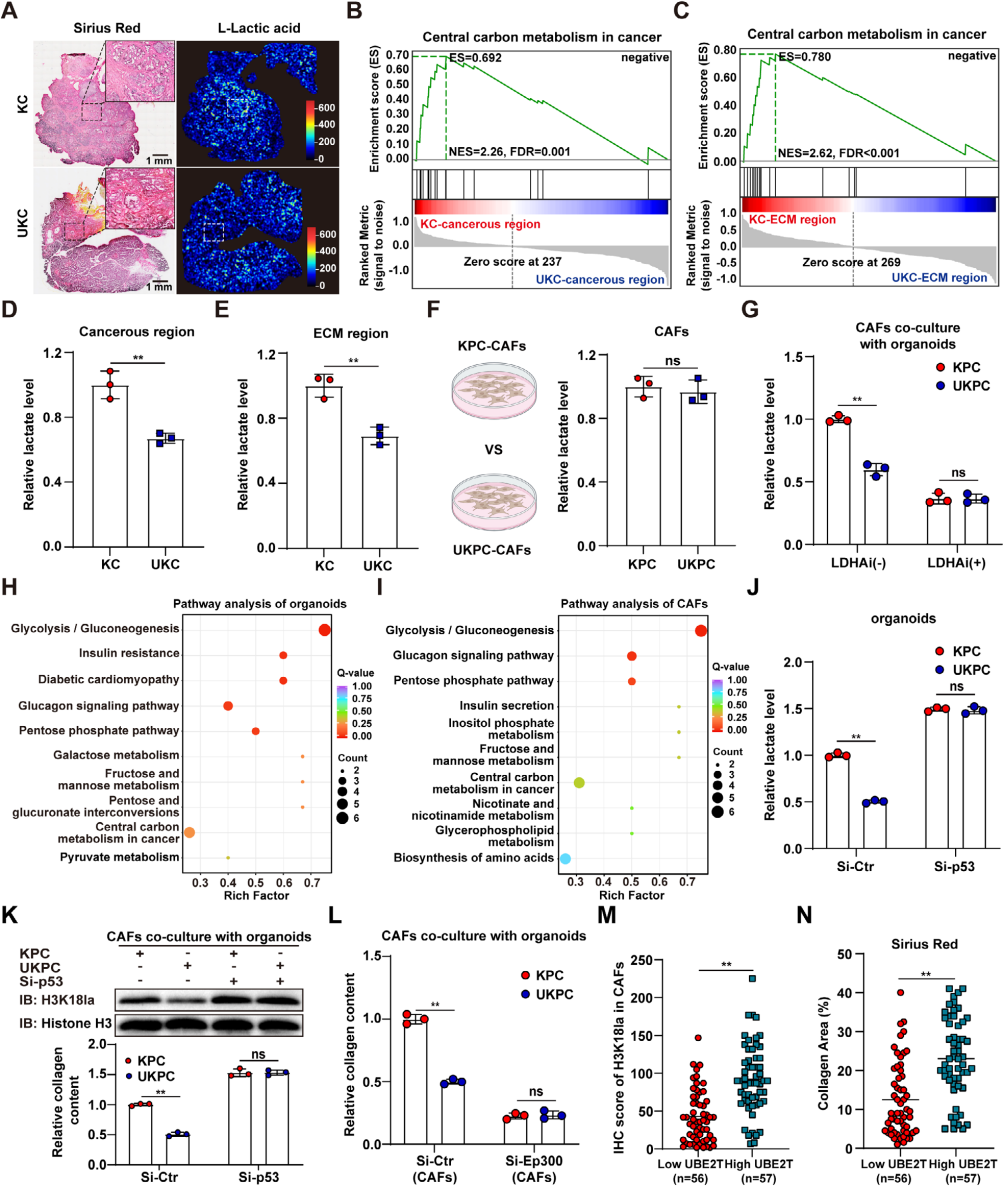
UBE2T‐p53 axis mediates lactate metabolic crosstalk by reprogramming glycolysis to promote stromal deposition. (A) Sirius Red staining and MSI of lactate in pancreatic tissues of KC and UKC mice. (B,C) GSEA of central carbon metabolism in cancerous (B) and ECM (C) regions with or without *Ube2t* deletion using spatial metabolomics data. (D,E) Lactate levels in cancerous (D) and ECM (E) regions with or without *Ube2t* deletion based on spatial metabolomics analysis (*n* = 3). (F) Relative lactate levels in CAFs with or without *Ube2t* deletion (*n* = 3). (G) Relative lactate levels in CAFs co‐cultured with KPC or UKPC organoids treated with or without oxamate (*n* = 3). (H,I) Metabolic pathway analysis of organoids with or without *Ube2t* deletion (H) and co‐cultured CAFs (I). (J) Relative lactate levels in CAFs co‐cultured with KPC and UKPC organoids with or without p*53* knockdown (*n* = 3). (K) Protein levels of H3K18la and relative collagen content (*n* = 3) in CAFs co‐cultured with KPC and UKPC organoids with or without *p53* knockdown. (L) Relative collagen content of Si‐Ctr and Si‐Ep300 CAFs co‐cultured with KPC or UKPC organoids(*n* = 3). (M,N) Quantification of H3K18la in CAFs(M) and Sirius Red staining (N) with low or high UBE2T expression. Student's *t* test in D–G, J–N, results are presented as the mean ± SD. ^**^ *p* < 0.01; ns, no significance.

To elucidate the regulatory role of UBE2T in lactate metabolism, we generated pancreatic conditional *Ube2t* knockout KC mice (UKC) and performed spatial metabolomic analysis on pancreatic tissues (Figure 4A). We observed decreased central carbon metabolism and reduced lactate accumulation in both the cancerous regions and ECM regions of pancreatic tissues from UKC mice (Figure 4B–E; Figure S5). To determine whether UBE2T directly regulates lactate production and collagen secretion in CAFs, we analyzed culture medium from KPC and UKPC CAFs. The results indicated *Ube2t* depletion in CAFs failed to reduce lactate production and collagen secretion (Figure 4F; Figure S6A). Co‐culture experiments revealed that CAFs exhibited significantly elevated lactate production and collagen secretion when cultured with KPC organoids compared to UKPC organoids, while pharmacological inhibition of LDHA in organoids completely abolished these differential secretion levels (Figure 4G; Figure S6B,C). To investigate the metabolic regulatory mechanism by which UBE2T promotes lactate production and collagen secretion in CAFs, we performed energy metabolomic profiling on KPC organoids, UKPC organoids, and co‐cultured CAFs (Figure S6D). Our analysis revealed that UBE2T simultaneously enhances glycolysis in both organoids and co‐cultured CAFs (Figure 4H,I; Figure S6E,F). Immunoblotting further showed that UBE2T upregulated the protein levels of key glycolytic enzymes (HK2, PFKFB3, PKM2, LDHA) in tumor cells, which was dependent on p53 regulation by UBE2T (Figure S6G). Subsequent co‐culture analysis revealed that UKPC organoids exhibited significantly reduced lactate secretion compared to KPC organoids, which was accompanied by decreased lactate and collagen secretion and reduced H3K18la levels in co‐cultured CAFs. However, UBE2T depletion no longer exerted these effects after *p53* knockdown in organoids (Figure 4J,K; Figure S6H). Furthermore, co‐culture of KPC and UKPC organoids with *Ep300*‐knockdown CAFs demonstrated that the regulatory effect of UBE2T on collagen secretion by CAFs is dependent on H3K18 lactylation (Figure 4L; Figure S6I). Analysis of clinical PDAC specimens demonstrated a significant positive correlation between tumor cell UBE2T and CAF H3K18la, and patients with high UBE2T expression displayed significantly enhanced stromal deposition (Figure 4M,N; Figure S6J–L). These findings suggest that the UBE2T‐p53 axis drives lactate metabolic crosstalk by rewiring glycolysis, thereby promoting stromal deposition.

### 2.5 UBE2T‐Driven Stromal Desmoplasia Impairs Anti‐PD‐1 Immunotherapy in PDAC

**FIGURE 5:**
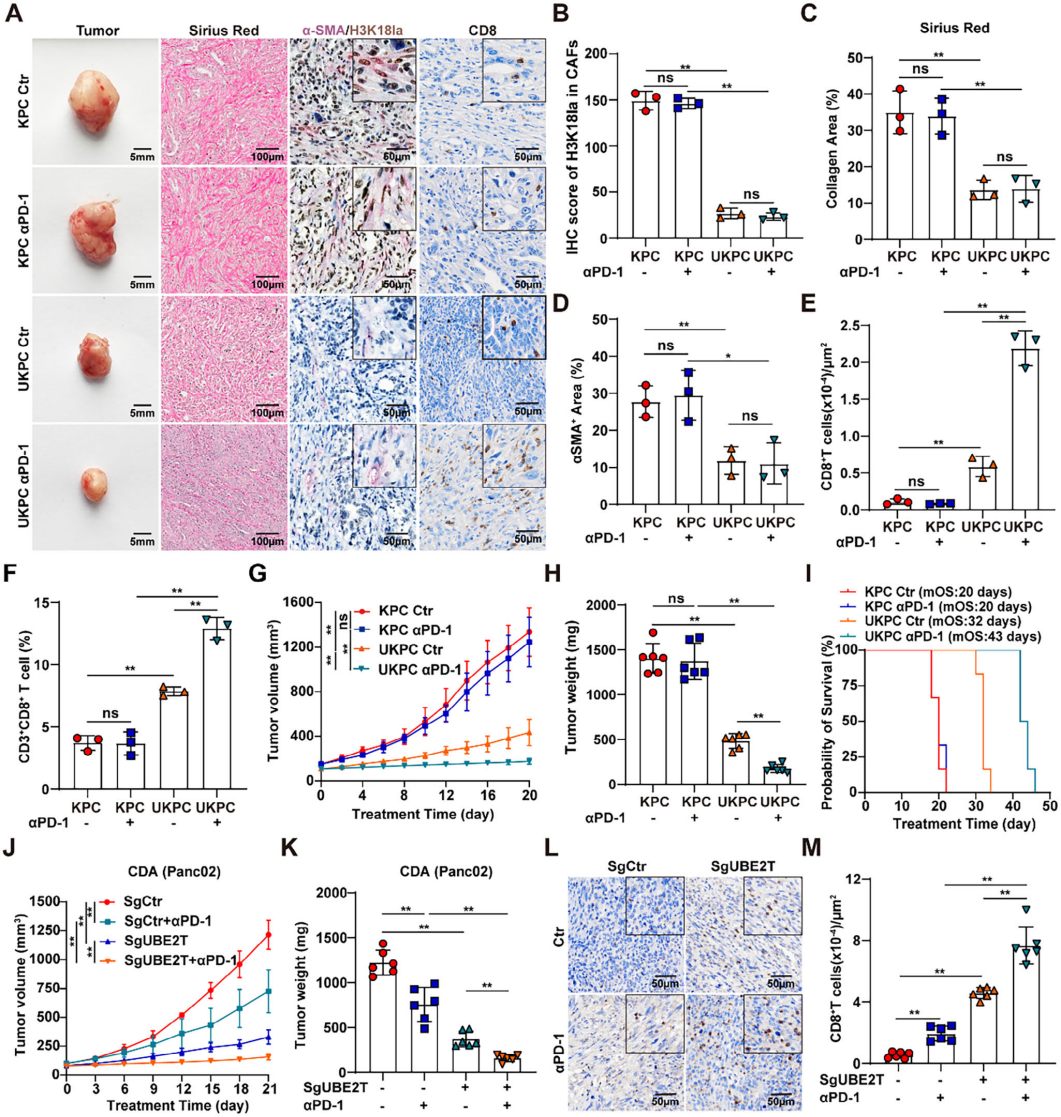
*Ube2t* depletion suppresses stromal deposition and improves anti‐PD‐1 therapy in PDAC. (A) Representative images of tumors and PDAC tissues stained with Sirius red, H3K18la/α‐SMA and CD8 in KPC and UKPC allografts treated with or without anti‐PD‐1 therapy. (B–E) Quantitative analysis of H3K18la/α‐SMA (B), Sirius red (C), α‐SMA (D), and CD8 (E) staining in KPC and UKPC allografts treated with or without anti‐PD‐1 therapy (*n* = 3). (F) Flow cytometric quantification of CD3^+^CD8^+^ T cells among CD45^+^ cells in KPC and UKPC allografts treated with or without anti‐PD‐1 therapy (*n* = 3). (G–I) Tumor growth (G), tumor weight (H) and OS (I) analysis of KPC and UKPC allografts treated with or without anti‐PD‐1 therapy (*n* = 6). (J–M) Tumor growth (J) and tumor weight (K) analysis, and representative images (L) and quantification (M) of CD8 staining in indicated genotypic Panc02 cells‐derived allografts treated with or without anti‐PD‐1 therapy (*n* = 6). One‐way ANOVA with Bonferroni correction in B–H,J,K and M, results are presented as the mean ± SD. ^*^ *p* < 0.05; ^**^ *p* < 0.01; ns, no significance.

To evaluate the regulatory role of UBE2T in the TME and its impact on immunotherapy efficacy, we established KPC and UKPC allograft models treated with anti‐PD‐1 antibody therapy. Genetic ablation of *Ube2t* resulted in reduced H3K18la levels in CAFs within tumor tissues (Figure 5A,B). Sirius red staining and α‐SMA immunofluorescence demonstrated that *Ube2t* knockout significantly reduced stromal deposition in the TME (Figure 5A,C,D; Figure S7A). Immunohistochemistry and flow cytometry analyses revealed that *Ube2t* deficiency enhanced CD8^+^ T cell infiltration within the TME, with this effect being markedly amplified when combined with anti‐PD‐1 antibody therapy (Figure 5A,E,F; Figure S7D). Moreover, the combination of *Ube2t* depletion with anti‐PD‐1 antibody significantly inhibited tumor growth and prolonged survival in PDAC mice (Figure 5G–I; Figure S7B,C). To further validate these findings, we evaluated the effect of UBE2T on anti‐PD‐1 antibody efficacy in a Panc02 cell‐derived allograft model. Consistent with previous findings, the combination of *Ube2t* knockout with anti‐PD‐1 therapy significantly inhibited tumor growth and enhanced CD8^+^ T cell infiltration in tumor tissues (Figure 5J–M; Figure S7E). These findings identify UBE2T as a critical regulator driving stromal desmoplasia and impairing immunotherapy efficacy in PDAC.

### 2.6 Pharmacological Targeting of UBE2T by PGG Inhibits Lactate Metabolic Crosstalk and Stromal Deposition

**FIGURE 6:**
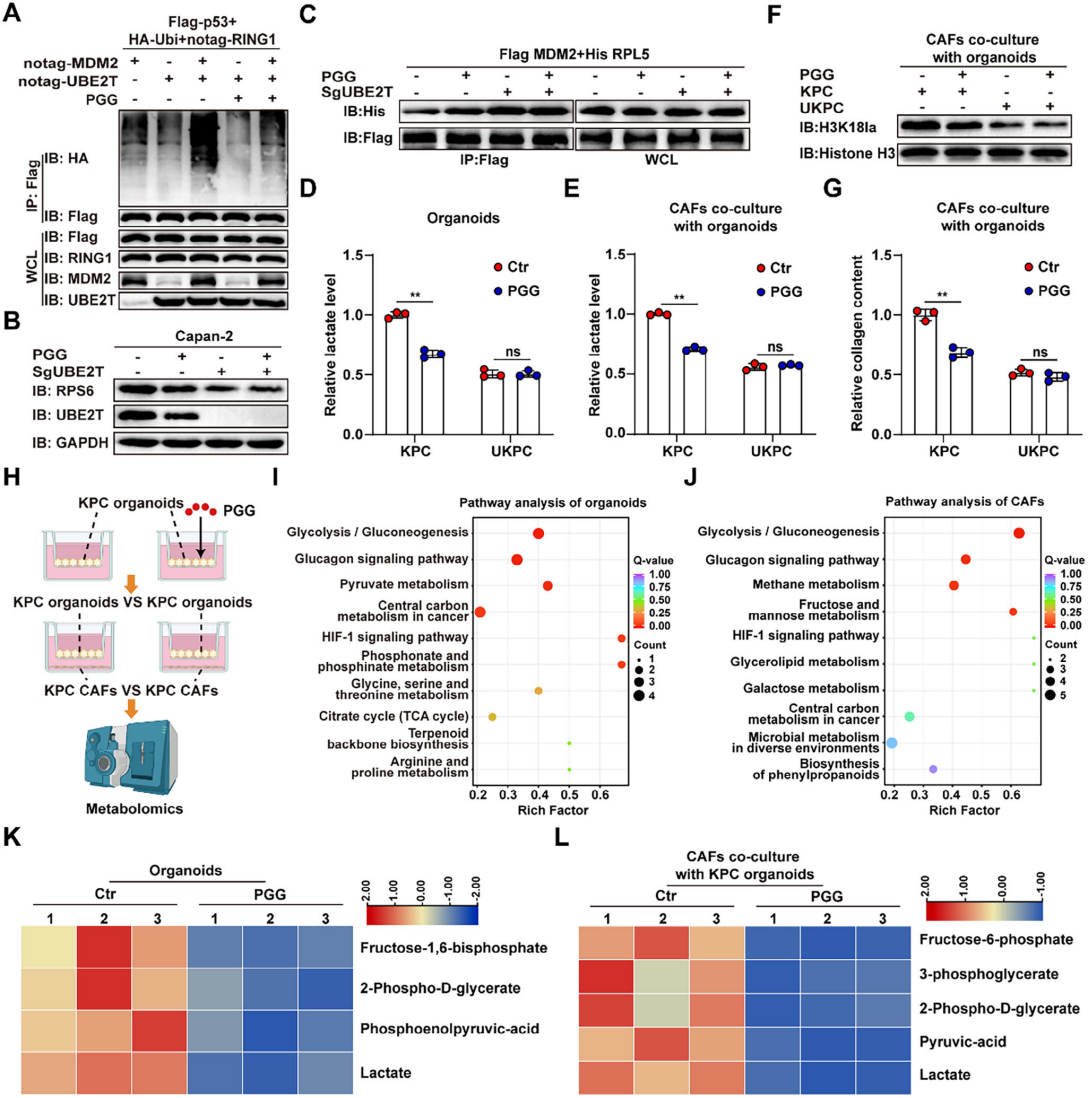
PGG inhibits lactate metabolic crosstalk and stromal deposition in PDAC. (A) Ubiquitination assay evaluating the effect of PGG on p53 ubiquitination in HEK‐293T cells co‐expressing the indicated plasmids. (B) Protein level of RPS6 in SgCtr and SgUBE2T Capan‐2 cells with or without PGG treatment (10 µm). (C) CoIP assays showing the interaction between MDM2 and RPL5 in SgCtr and SgUBE2T Capan‐2 cells with or without PGG treatment. (D,E) Relative lactate levels of KPC and UKPC organoids treated with or without PGG (10 µm) (D) and co‐cultured CAFs (E) (*n* = 3). (F) Protein level of H3K18la in CAFs co‐cultured with KPC and UKPC organoids treated with or without PGG. (G) Relative collagen content in CAFs co‐cultured with KPC and UKPC organoids with or without PGG treatment (*n* = 3). (H) Schematic diagram of energy metabolomics analysis after PGG treatment. (I,J) Metabolic pathway analysis of organoids with or without PGG treatment (I) and co‐cultured CAFs (J). (K,L) Differential metabolites of glycolysis in organoids with or without PGG treatment (K) and co‐cultured CAFs (L) detected by energy metabolomics (*n* = 3). Student's *t* test in D, E and G, results are presented as the mean ± SD. ^**^ *p* < 0.01.

Our previous study identified pentagalloylglucose (PGG) as a pharmacological inhibitor of UBE2T [26]. Here, we demonstrate that PGG treatment, by inhibiting UBE2T, disrupts ribosome biogenesis, enhances RPL5‐MDM2 binding, and consequently inhibits the UBE2T‐driven positive feedback loop that promotes p53 degradation (Figure 6A–C; Figure S8A–C). We next examined the effects of PGG‐treated KPC and UKPC organoids on co‐cultured CAFs. The results showed that PGG treatment simultaneously reduced lactate production in both KPC organoids and co‐cultured CAFs (Figure 6D,E). Furthermore, co‐cultured CAFs exhibited decreased H3K18la levels and reduced collagen secretion (Figure 6F,G). In contrast, PGG did not show any additional effects when UKPC organoids were co‐cultured with CAFs. To elucidate the mechanism by which PGG regulates the crosstalk of lactate metabolism between pancreatic cancer cells and CAFs, we performed energy metabolomic profiling on both control and PGG‐treated KPC organoids and their co‐cultured CAFs (Figure 6H). The analysis revealed that PGG significantly reduced glycolytic intermediates and lactate levels in KPC organoids and co‐cultured CAFs by inhibiting glycolysis (Figure 6I–L). Immunoblotting further revealed that PGG inhibited the expression of glycolytic enzymes in tumor cells by targeting UBE2T (Figure S8D). These findings suggest that PGG can target UBE2T to inhibit glycolysis, thereby attenuating lactate‐mediated metabolic crosstalk and stromal deposition in PDAC.

### 2.7 The Combination of PGG and Anti‐PD‐1 Exhibits Potently Therapeutic Efficacy in PDAC

**FIGURE 7:**
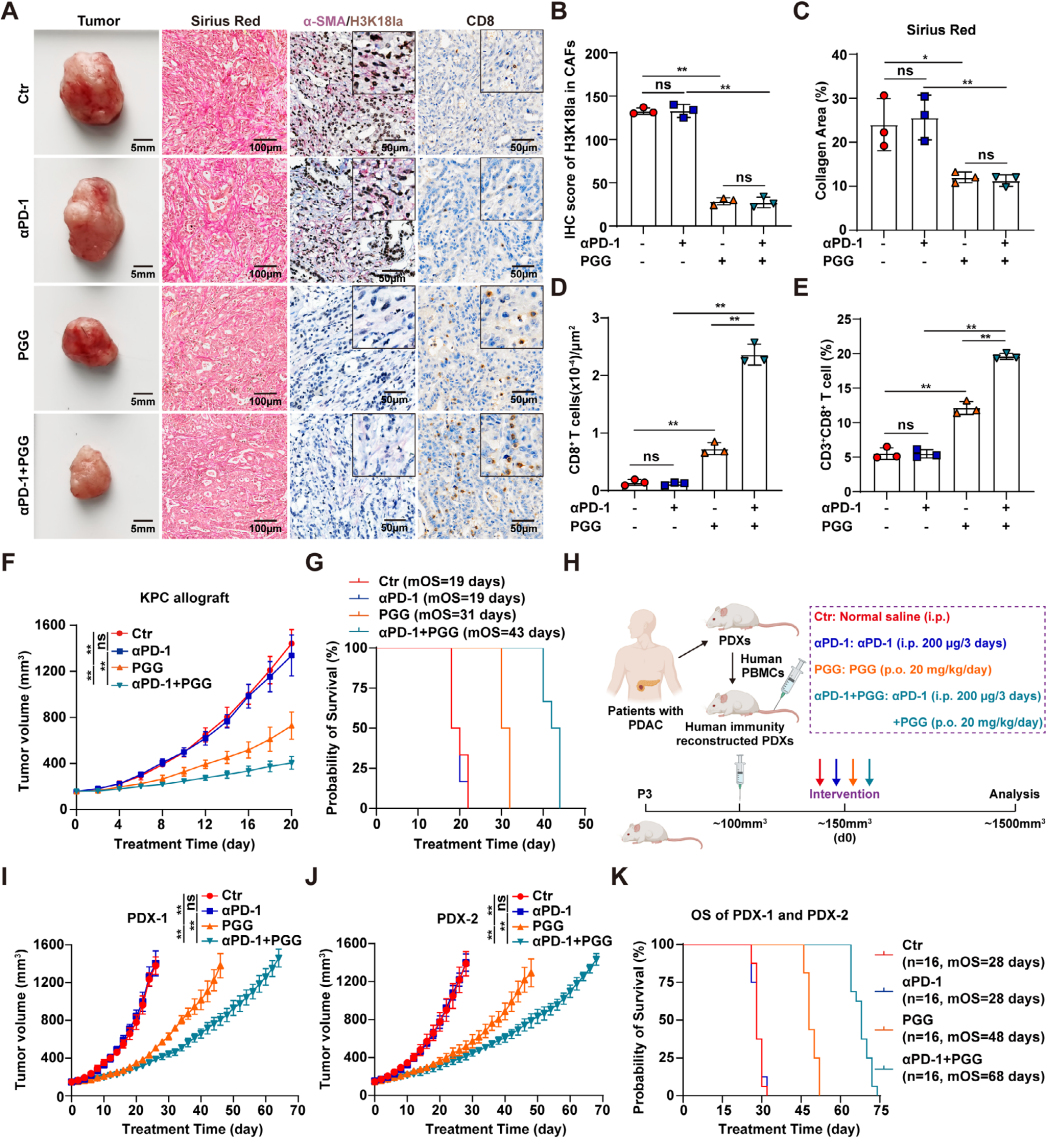
PGG improves the therapeutic efficacy of anti‐PD‐1 antibody in PDAC. (A–D) Representative images of tumors and PDAC tissues stained with Sirius red, H3K18la/α‐SMA and CD8 (A), and quantification of H3K18la/α‐SMA (B), Sirius Red (C), and CD8^+^ T cells (D) in KPC allografts treated with or without PGG and/or anti‐PD‐1 therapy (*n* = 3). (E) Flow cytometric quantification of CD3^+^CD8^+^ T cells among CD45^+^ cells in KPC allografts treated with or without PGG and/or anti‐PD‐1 therapy (*n* = 3). (F,G) Tumor growth (F) and OS (G) analysis of KPC allografts treated with or without PGG and/or anti‐PD‐1 therapy (*n* = 6). (H) Schematic diagram of the treatment of PGG and/or anti‐PD‐1 antibody in human immunity reconstructed PDXs. (I,J) Tumor growth of PDX‐1 (I) and PDX‐2 (J) treated with or without PGG and/or anti‐PD‐1 therapy (*n* = 8). K) OS analysis of PDXs treated with or without PGG and/or anti‐PD‐1 therapy. One‐way ANOVA with Bonferroni correction in B–F,I and J, results are presented as the mean ± SD. ^*^ *p* < 0.05; ^**^ *p* < 0.01; ns, no significance.

To evaluate the in vivo toxicity of PGG, KM mice were orally administered with PGG. The results showed that PGG did not significantly affect body weight or cause any obvious organ toxicity, indicating that PGG has favorable biosafety (Figure S9). To investigate the effects of PGG on the PDAC TME and immunotherapy efficacy, we treated KPC allograft mice with PGG and anti‐PD‐1 antibody. Our results showed that PGG suppressed H3K18la levels in CAFs and reduced stromal deposition (Figure 7A–C; Figure S10A,B). PGG monotherapy promoted CD8^+^ T cell infiltration in tumor tissues, with enhanced effects when combined with anti‐PD‐1 antibody (Figure 7A,D,E; Figure S10D). Furthermore, the combination of PGG and anti‐PD‐1 antibody significantly inhibited PDAC tumor progression (Figure 7F; Figure S10C,E). We evaluated the combination index of PGG and anti‐PD‐1 antibody using CombPDX [29] and found a marked synergistic effect in the KPC model (Figure S10F). Next, we assessed the long‐term survival impact of this combination in KPC allograft mice and patient‐derived xenograft (PDX) models from two patients. The combination therapy significantly prolonged survival in the KPC model (median overall survival [mOS], 19 days vs. 43 days) (Figure 7G; Figure S10G). To evaluate the efficacy of PGG combined with anti‐PD‐1 antibody in humanized models, we reconstituted the immune system of PDX model via tail vein infusion of peripheral blood mononuclear cells (PBMCs) and administered corresponding drug interventions (Figure 7H). The results demonstrated that the combination therapy significantly extended overall survival in both PDX‐1 and PDX‐2 models (mOS, 28 days vs. 68 days) (Figure 7I–K; Figure S11). These findings suggest that PGG functions as a promising immunotherapeutic potentiator for PDAC.

## 3 Discussion

Accumulating evidence substantiates that aerobic glycolysis and lactylation critically potentiate malignant phenotypes of cancer [16, 30, 31]. Nevertheless, the mechanism by which glycolysis‐driven lactylation regulates stromal deposition in PDAC remains to be fully elucidated. Here, our investigation reveals that UBE2T facilitates glycolytic rewiring and lactate metabolic crosstalk by amplifying p53 positive feedback degradation, thereby inducing lactylation in CAFs and promoting a dense stromal microenvironment in PDAC. Furthermore, targeting UBE2T disrupts tumor cell‐CAF metabolic crosstalk, reduces stromal deposition, enhances CD8^+^ T cell infiltration, and improves the therapeutic efficacy of anti‐PD‐1 antibody.

Lactylation, an emerging lactate‐mediated post‐translational modification (PTM), orchestrates diverse oncogenic processes including malignant progression, immune evasion, and therapeutic resistance in cancer [17, 18, 19, 32, 33]. Here, our study reveals that lactate‐mediated upregulation of H3K18la in CAFs promotes a stroma‐dense TME. Previous studies have demonstrated that H3K18la facilitates tissue fibrosis and contributes to the immunosuppressive microenvironment of PDAC [20, 33, 34]. We similarly observed that LDHA inhibition attenuates stromal deposition and enhances anti‐tumor immunity in PDAC. Therefore, targeting lactylation‐driving factors unveils novel therapeutic opportunities for PDAC. Numerous studies have demonstrated that p53 directly regulates glycolysis and lactate production by suppressing glucose transporters or metabolic enzymes, while also serving as a key modulator of the TME [24, 25, 35, 36, 37, 38, 39]. Our study further revealed that UBE2T enhances lactate production and elevates H3K18la levels in CAFs, leading to increased collagen secretion, which is dependent on p53 degradation in tumor cells. These findings implicate UBE2T‐mediated p53 degradation as a critical event promoting lactylation‐mediated stromal deposition and immunosuppression in PDAC.

Our previous study demonstrated that UBE2T mediates K48‐linked ubiquitination of p53 and its subsequent degradation [26]. As a negative regulator of ribosome biogenesis, p53 is stabilized upon impaired ribosome biogenesis due to enhanced binding of ribosomal proteins to MDM2 [27, 28, 40]. Based on these findings, we hypothesized that UBE2T mediates p53 ubiquitination and degradation through a feedback regulatory mechanism. Our results demonstrate that UBE2T acts as an initiator that promotes MDM2‐mediated p53 degradation by regulating ribosome biogenesis, thereby establishing a self‐reinforcing positive feedback loop. Previous work established aberrant overexpression of UBE2T across malignancie, which drives cancer progression, metastasis, and therapy resistance [26, 41, 42, 43, 44, 45]. UBE2T has been implicated in glycolytic regulation and proposed as a predictive biomarker for immunotherapy sensitivity [46, 47]. Our study further reveals that UBE2T enhances H3K18 lactylation in CAFs by regulating p53 degradation to reprogram glycolysis, thereby driving stromal fibrosis and restricting CD8^+^ T cell infiltration in PDAC. In addition, our findings indicate that targeting UBE2T, which modulates MDM2‐mediated p53 ubiquitination and degradation, exhibits tumor‐suppressive function in KPC models. Consistent with this observation, prior studies have reported that suppressing MDM2‐mediated p53 degradation can effectively inhibit tumor progression and metastasis in KPC models [48]. These observations may be attributed to the preserved function of wild‐type p53 in PDAC. Future studies are necessary to further investigate the regulatory mechanisms by which UBE2T modulates both wild‐type p53 and p53 mutants harboring mutations at different sites.

From a clinical perspective, our study proposes a synergistic immunotherapy strategy for PDAC. Immune checkpoint inhibitors (ICI) represented by anti‐PD‐1 antibody exhibit therapeutic efficacy only in specific PDAC subtypes, largely attributable to tumor‐intrinsic metabolic reprogramming and the immunosuppressive TME [49, 50]. PGG, a highly selective and low‐toxicity UBE2T inhibitor, has previously shown potent antitumor activity across multiple malignancies [26, 42, 51]. Furthermore, PGG modulates glycolytic enzyme expression and immune cell infiltration in tumor tissue [52, 53]. Here, our study demonstrates that targeting UBE2T with PGG disrupts lactate‐mediated metabolic crosstalk between tumor cells and CAFs via glycolysis inhibition, thereby alleviating stromal desmoplasia, enhancing intratumoral CD8^+^ T cells infiltration, and synergizing with anti‐PD‐1 therapy. Notably, our findings highlight translational potential of PGG as an immunotherapeutic sensitizer. In KPC allograft and immune‐reconstituted PDX models, the combination of PGG and anti‐PD‐1 therapy conferred significant survival benefits. Further clinical investigations are warranted to validate PGG‐ICI combination regimens as a novel therapeutic paradigm for PDAC.

In summary, our study reveals that UBE2T promotes a positive feedback loop of p53 degradation by regulating ribosome biogenesis, thereby enhancing glycolysis and inducing H3K18 lactylation in CAFs, ultimately driving tumor microenvironment remodeling in PDAC. Notably, the combination of PGG with anti‐PD‐1 therapy demonstrates marked synergistic efficacy and survival benefits, highlighting the potential clinical utility of this strategy to improve immunotherapy in PDAC.

## 4 Experimental Section

### 4.1 Human Clinical Specimens

This study collected clinical specimens of 113 PDAC patients who had not received preoperative radiotherapy, chemotherapy, or immunotherapy from the Second Hospital of Lanzhou University. Surgical specimens from two patients with PDAC were used to establish patient‐derived xenografts (PDX) models. Informed consent was obtained from all the patients. This study was carried out in accordance with the Declaration of Helsinki and was approved by the Medical Ethics Review Committee of Lanzhou University Second Hospital (approval number: 2024A‐074).

### 4.2 Organoids Studies

KPC/UKPC organoids were generated from pancreatic tumors of KPC/UKPC mice. The tumors were minced and digested at 37°C for 30–60 min in medium consisted of Advanced DMEM/F‐12 (#12634010, Gibco, New York, USA), 2 mm GlutaMAX Supplement (#35050061, Gibco, New York, USA), 10.5 µm Y‐27632 (#HY‐10071, MCE, New Jerse, USA), 500 nm A83‐01 (#HY‐10432, MCE, New Jerse, USA), 100 µg mL^−1^ Primocin (#ant‐pm‐05, InvivoGen, Toulouse, FR), and 1 mg mL^−1^ collagenase XI (#C7657, Sigma–Aldrich, Missouri, USA). The mixture was filtered using a 70 µm cell strainer (#352350, Corning, New York, USA) to obtain the cell suspension. After centrifugation, the precipitate containing adult stem cells was collected and resuspended in Cultrex UltiMatrix Reduced Growth Factor Basement Membrane Extract (#BME001‐05, Bio‐Techne, Minnesota, USA). The mixtures were planted in 24‐well plates and cultured in PancreaCult Organoid Growth Medium (Mouse) (#06040, STEMCELL Technologies, Vancouver, Canada).

### 4.3 Generation of Transgenic Mice

*LSL‐Kras^G12D/+^* mice (RRID: IMSR_NM‐KI‐190003), *LSL‐Trp53^R172H/+^* mice (RRID: IMSR_NM‐KI‐220071) and *Ube2t ^flox/flox^* mice (RRID:IMSR_NM‐CKO‐2115005) were obtained from Shanghai Model Organisms Center, Inc. (Shanghai, China). *Pdx1‐Cre* mice (RRID: IMSR_JAX:014647) were purchased from The Jackson Laboratory (Maine, USA). These mice were interbred to generate *LSL‐Kras^G12D/+^/ Pdx1‐Cre* (KC), *LSL‐Kras^G12D/+^/LSL‐Trp53^R172H/+^/ Pdx1*‐*Cre* (KPC), *Ube2t ^flox/flox^*‐KC (UKC) and *Ube2t ^flox/flox^*‐KPC (UKPC) mice. To ascertain the genotype, tail tissue from one‐month‐old mice was obtained and minced. DNA samples were prepared using the Mouse Direct PCR Kit (For Genotyping) (#B40015, Selleck Chemicals, Texas, USA). The detection of the genotypes was performed with Agarose Gel Electrophoresis and DNA sequencing. DNA sequencing confirmed that KPC mice harbor a heterozygous *Trp53* mutation (*Trp53^R172H/WT^*), thus retaining one wild‐type Trp53 allele.

### 4.4 KPC Mouse‐Derived Allografts Study

The tumors from KPC and UKPC mice were cut into small pieces about 3 mm in diameter. Then the tissue pieces were transplanted subcutaneously into 6‐week‐old female C57BL/6JGpt mice (#N000013, GemPharmatech, Nanjing, China, RRID: IMSR_GPT: N000013) to establish KPC and UKPC allografts. When the tumor volume reached 1000 mm^3^, the tumors was minced and transplanted to newly acquired mice. The third‐generation tumors are obtained for drug evaluation experiments. For drug intervention studies, anti‐Mouse PD‐1 (#P372, Leinco Technologies, Missouri, USA, RRID: AB_2749820) was intraperitoneally administered at a dosage of 200 µg every 3 days, and sodium oxamate (#HY‐W013032A, MCE, New Jerse, USA) was intraperitoneally administered at a dosage of 750 mg kg^−1^ every day. PGG (#HY‐N0527, MCE, New Jerse, USA) was orally administered at a dosage of 20 mg kg^−1^ every day. The mice with tumors about 150 mm^3^ were grouped randomly to receive drug treatments. In the short‐term tumor progression assessment, all mice were euthanized once any one tumor reached a volume of 1,500 mm^3^. Three randomly selected mice per group were used for staining analysis, and the remaining three mice were used for flow cytometry analysis. In the long‐term survival assessment, the ethical endpoint was defined as each tumor volume approaching 1,500 mm^3^. The sizes of tumors were measured every two days. All animal experiments were approved by the Laboratory Animal Ethics Committee of Lanzhou University Second Hospital (approval number: D2024‐055).

### 4.5 PDX Study

NOD/ShiLtJGpt‐Prkdc^em26Cd52^Il2rg^em26Cd22^/Gpt mice (#T001475, GemPharmatech, Nanjing, China, RRID: IMSR_GPT: T001475) were used to establish PDX models. Surgical specimens from patients with pancreatic cancer were cut into pieces of approximately 3 mm in diameter, embedded in high‐concentration basement membrane matrix (#354248, Corning, New York, USA), and implanted subcutaneously into the axilla of 6‐week‐old female mice. When the volume reached approximately 1000 mm^3^, the tumors were dissected and transplanted to the next batch of mice, and tumors from the third batch of mice were obtained for drug evaluation experiments. In order to explore the effect of PGG on immunotherapeutic efficacy in PDX models, the human peripheral blood mononuclear cells (PBMCs) (SAILYBIO, Shanghai, China) were injected intravenously at a cell count of 5 × 10^6^ to reconstitute the immune system when the tumor volume reached about 100 mm^3^. The PBMCs used in this study were isolated from the peripheral blood of healthy female donors under ethical approval (ID: 2024A‐074). PGG (#HY‐N0527, MCE, New Jerse, USA) was orally administered at a dosage of 20 mg kg^−1^ every day whereas Sindilizumab (Innovent Bio, Suzhou, China) was intraperitoneally administered at a dosage of 200 µg every 3 days. When the tumor volume reached 150 mm^3^, the mice were randomly divided into groups for drug intervention. The tumor size was measured every two days, with a tumor volume of 1500 mm^3^ serving as the ethical endpoint.

### 4.6 Cell‐Derived Allograft Model Study

Six‐week‐old female C57BL/6JGpt mice (#N000013, GemPharmatech, Nanjing, China, RRID: IMSR_GPT: N000013) were used to establish the cell‐derived allograft models. Panc02 cells with different UBE2T genotypes (SgCtr, SgUBE2T) were mixed separately with PBS and Matrigel (#354234, Corning, New York, USA) and subcutaneously injected into the axilla of mice at a dose of 5 × 10^6^ cells. When tumor volume reached 100 mm^3^ the mice were divided into four groups: SgCtr, SgCtr plus anti‐mouse PD‐1 (intraperitoneal, 200 µg every 3 days), SgUBE2T, SgUBE2T plus anti‐mouse PD‐1. Tumor size was measured every three days, and all mice were euthanized as any one tumor volume reaching 1500 mm^3^.

### 4.7 Statistical Analysis

The statistical analyses in this study were all performed by SPSS 27.0 (IBM, Armonk, New York, USA). The Shapiro‐Wilk test was used to assess normality of the data. For normally distributed data, Student's t‐test and one‐way analysis of variance (ANOVA) were used to compare differences between two and multiple groups. Post‐hoc comparisons following ANOVA were adjusted for multiple testing using Bonferroni correction. Nonparametric tests were used to analyze data that are not normally distributed. Kaplan‐Meier curves with Log‐rank tests were used to describe survival differences. All tests were two sided and *p* values <0.05 were considered statistically significant. Values are presented as the mean ± standard deviation (SD) for normally distributed data. All assays were independently repeated at least three times biologically. The “n” values refer to the number of independent biological replicates per group.

Additional details of the materials and methods can be found in Supplementary methods.

## Author Contributions

Y.M., W.L. and M.L. contributed equally to this work; Z.J., X.J. and Y.M. conceived and designed the research; Y.M., W.L., M.L, T.W., and H.S. performed in vivo experiments; Y.M., W.L., M.L., B.Z., H.Q. and Y.D. performed in vitro experiments; Y.M., X.J. and Q.H. contributed to the bioinformatics analysis; Y.M. and K.W. contributed to the statistical analysis; X.G. and L.Q. provide pathology evaluations; H.Z. and Z.Y. contributed to the collection of clinical specimens; Y.M., W.L. and M.L. analysed and interpreted the results of the study; W.S., Z.Y., X.J. and Z.J. supervised the study; Y.M. and Z.J. wrote the manuscript; W.S., Z.Y., X.J. and Z.J. provided a critical review of the manuscript; Z.J., Y.M., X.J. and Z.Y. provided funding of the study.

## Conflicts of Interest

The authors declare no conflict of interest.

## Supporting information




**Supporting File**: advs74317‐sup‐0001‐SuppMat.docx.

## Data Availability

The data that support the findings of this study are available from the corresponding author upon reasonable request.
