# Supplementary material for: UBE2T‐Driven p53 Degradation Rewires Glycolysis to Orchestrate Lactylation‐Mediated CAFs Activation and ECM Deposition in Pancreatic Cancer
Source: Adv Sci (Weinh). 2026 Feb 15;13(22):e14933. doi: 10.1002/advs.202514933 (PMC13088301; doi:10.1002/advs.202514933)
Supplement: Supplementary file 1 — Supporting File: advs74317‐sup‐0001‐SuppMat.docx. [file ADVS-13-e14933-s001.docx]

**Title: UBE2T-driven p53 degradation rewires glycolysis to orchestrate lactylation-mediated CAFs activation and ECM deposition in pancreatic cancer**

**Authors:**

Yong Ma^1,2,3#^, Wenbo Liu^1,2,3#^, Mingdou Li^1,2,3#^, Tao Wang^1,2,3,^ Bin Zhao^1,2,3,^ Keshen Wang^4^, Qichen He^1,2,3^, Haonan Sun^1,2,3^, Huiguo Qing^1,2^, Xiaoying Guan^5^, Wengui Shi^3,6^, Long Qin^3,6^, Yuman Dong^3,6^, Huinian Zhou^2,3^, Zeyuan Yu^2,3*^, Xiangyan Jiang^1,2,3*^, Zuoyi Jiao^2,3*^

**Supplementary Figures**

**
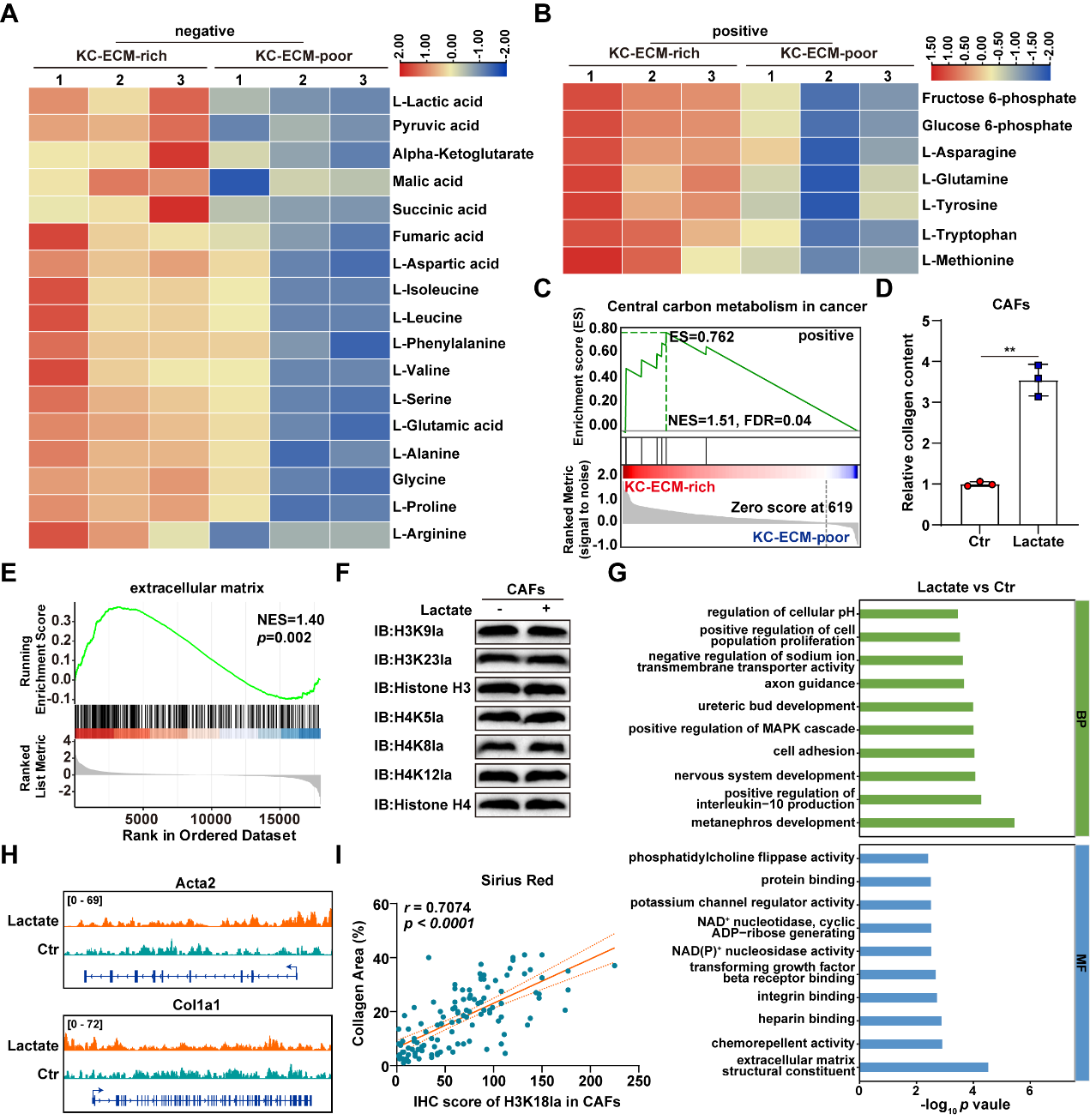
**

**Figure S1.** Lactate-mediated H3K18 lactylation in CAFs promotes stromal deposition. (A, B) Differential metabolites of center carbon metabolism in ECM-rich and ECM-poor regions detected by spatial metabolomics analysis in negative (A) and positive (B) condition (*n* = 3). (C) Gene Set Enrichment Analysis (GSEA) of central carbon metabolism in ECM-rich and ECM-poor regions using spatial metabolomics data. (D) Relative collagen content in CAFs with or without lactate treatment (10 mm) (*n* = 3). (E) Gene Ontology (GO) analysis of extracellular matrix in CAFs with or without lactate treatment (10 mm). (F) Protein levels of the indicated proteins in CAFs with or without lactate treatment (10 mm). (G) GO analysis of genes associated with H3K18la binding peaks in CAFs with or without lactate treatment. (H) Normalized read densities for H3K18la at the α-SMA and Col1a1 genes. (I) Correlation analysis between H3K18la of CAFs and collagen area in PDAC clinical samples. Student's *t* test in D, results are presented as the mean ± SD. ^**^*p* < 0.01.


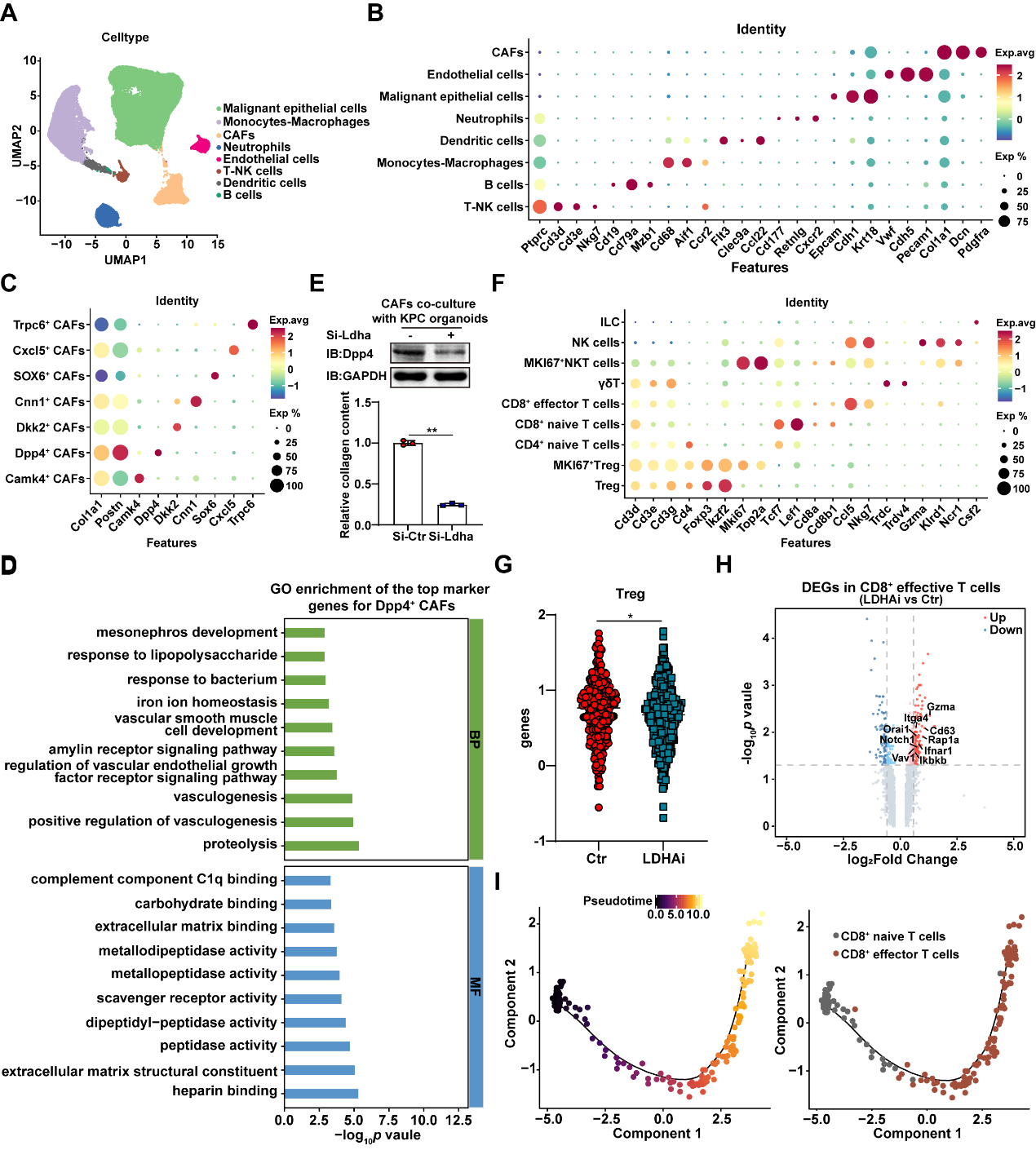


**Figure S2.** LDHA inhibition potentiates anti-tumor immune responses. (A) Uniform manifold approximation and projection (UMAP) plots from single-cell sequencing (scRNA-seq) data showing cell type annotations in KPC allografts treated with or without oxamate. (B) Markers of cell identification for major cell classes. (C) Markers of cell identification for CAFs subsets. (D) GO analysis of Dpp4^+^ CAFs based on top 100 marker genes. (E) Protein levels of Dpp4 and relative collagen content (*n* = 3) in CAFs co-cultured with KPC organoids with or without *Ldha* knockdown. (F) Markers of cell identification for T-NK cells subsets. (G) Immunosuppression score in Tregs from KPC allografts with or without oxamate analyzed by scRNA-seq. (H) Differentially expressed genes (DEGs) in CD8^+^ effective T cells from KPC allografts with or without oxamate analyzed by scRNA-seq. (I) Pseudotime distribution of CD8^+^ T cells. Student's *t* test in E and G, results are presented as the mean ± SD. ^*^*p* < 0.05, ^**^*p* < 0.01.
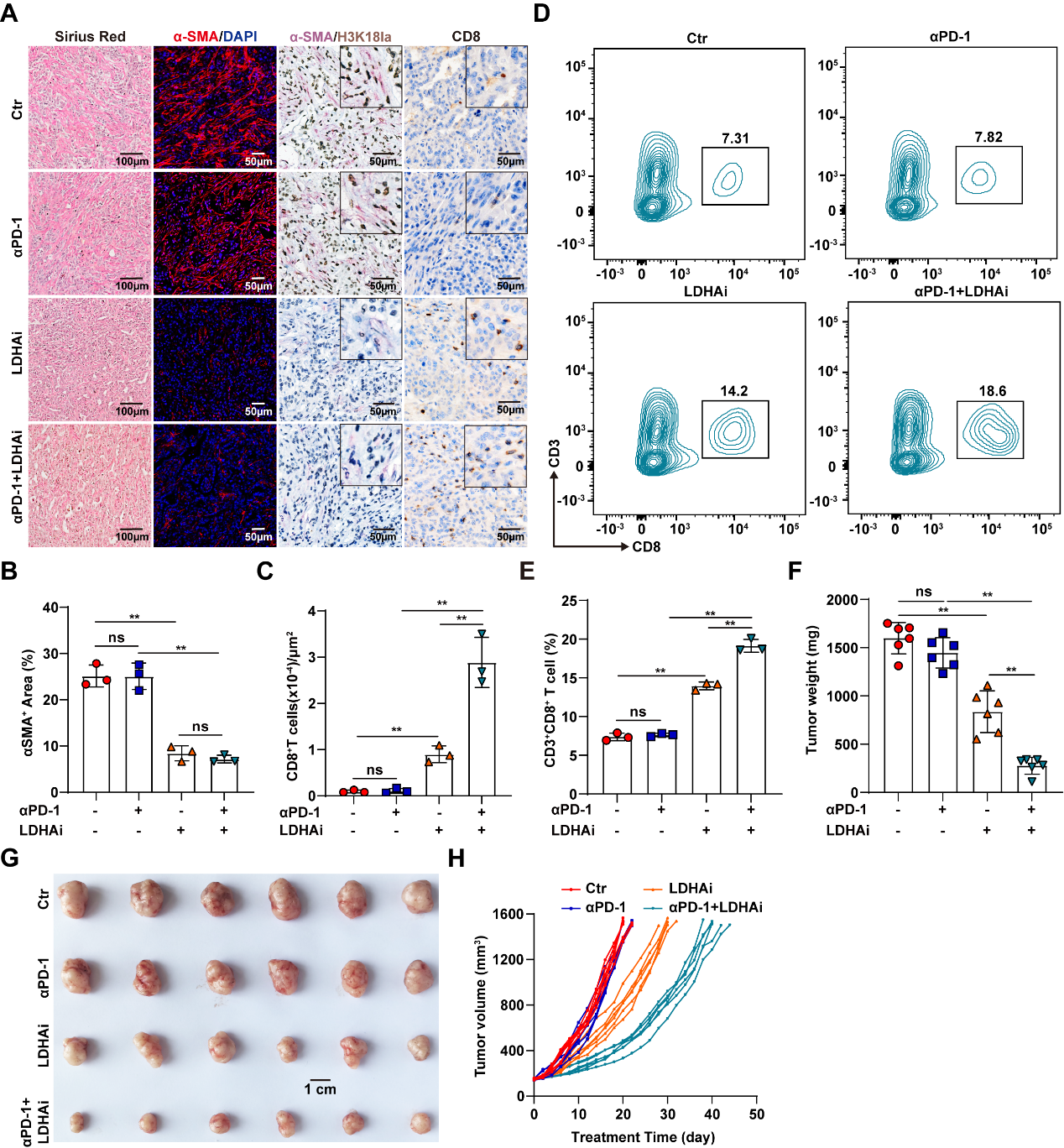


**Figure S3.** LDHA inhibition improves anti-PD-1 therapy in PDAC. (A-C) Representative images of tumors and PDAC tissues stained with Sirius red, α-SMA, H3K18la/α-SMA and CD8 (A), and quantification of α-SMA (B) and CD8 (C) in KPC allografts treated with or without oxamate and/or anti-PD-1 therapy (*n* = 3). (D, E) Representative flow cytometric analysis (FCA) images (D) and quantification (E) of CD3^+^CD8^+^ T cells among CD45^+^ cells in KPC allografts with or without oxamate and/or anti-PD-1 therapy (*n* = 3). (F, G) Quantification of tumor weight (F) and representative images of tumors (G) in KPC allografts with or without oxamate and/or anti-PD-1 therapy (*n* = 6). (H) Tumor growth of KPC allografts treated with or without oxamate and/or anti-PD-1 therapy (*n* = 6). One-way ANOVA with Bonferroni correction in B, C, E and F, results are presented as the mean ± SD. ^**^*p* < 0.01; ns, no significance.


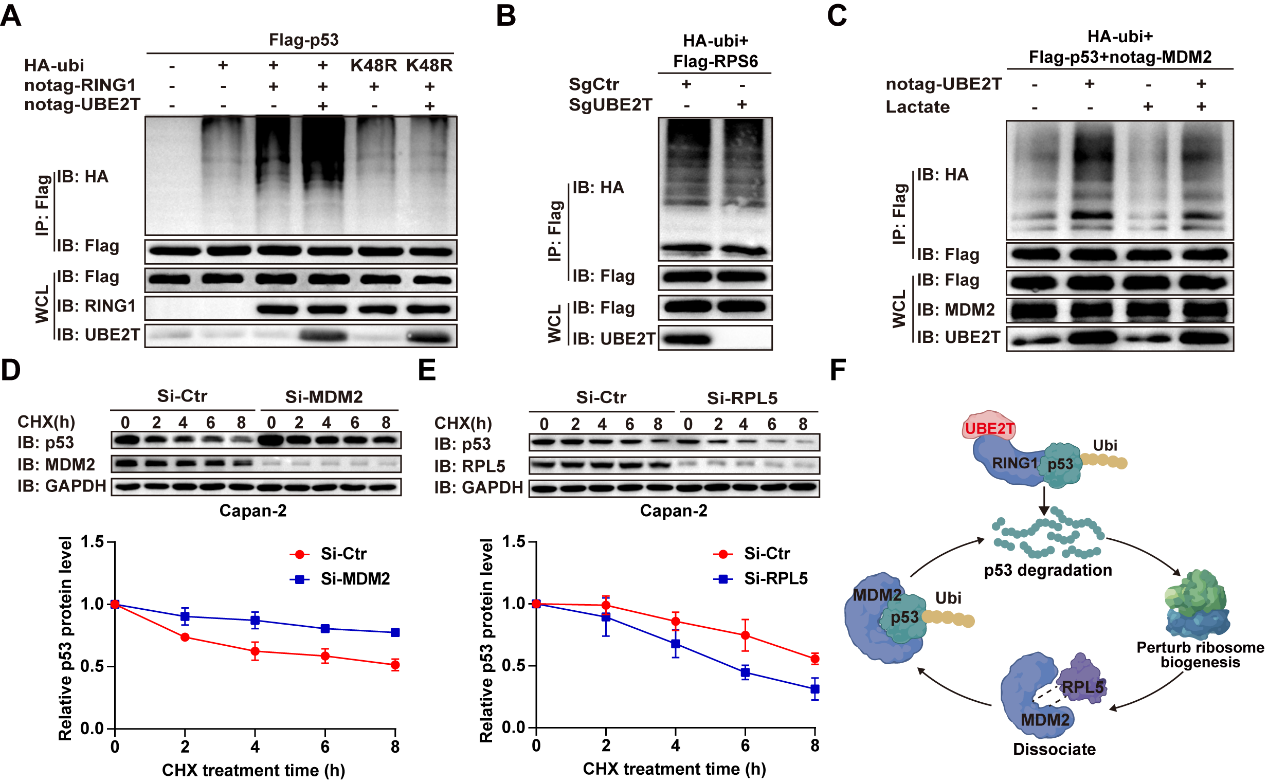


**Figure S4.** UBE2T potentiates MDM2-mediated p53 degradation. (A) Ubiquitination assay showing the degree of p53 ubiquitination in 293T cells expressing the indicated plasmids. (B) Ubiquitination assay showing the degree of RPS6 ubiquitination in SgCtr and SgUBE2T Capan-2 cells expressing the indicated plasmids. (C) Ubiquitination assay showing the degree of p53 ubiquitination in Capan-2 cells expressing the indicated plasmids. Cells were treated with or without lactate (10 mm). (D, E) Immunoblotting assay shows the half-life of p53 proteins treated with 100 ug mL^-1^ cycloheximide (CHX) (*n* = 3) in Capan-2 cells with or without *MDM2/RPL5* knockdown. (F) Schematic diagram of the p53 positive feedback degradation mechanism.


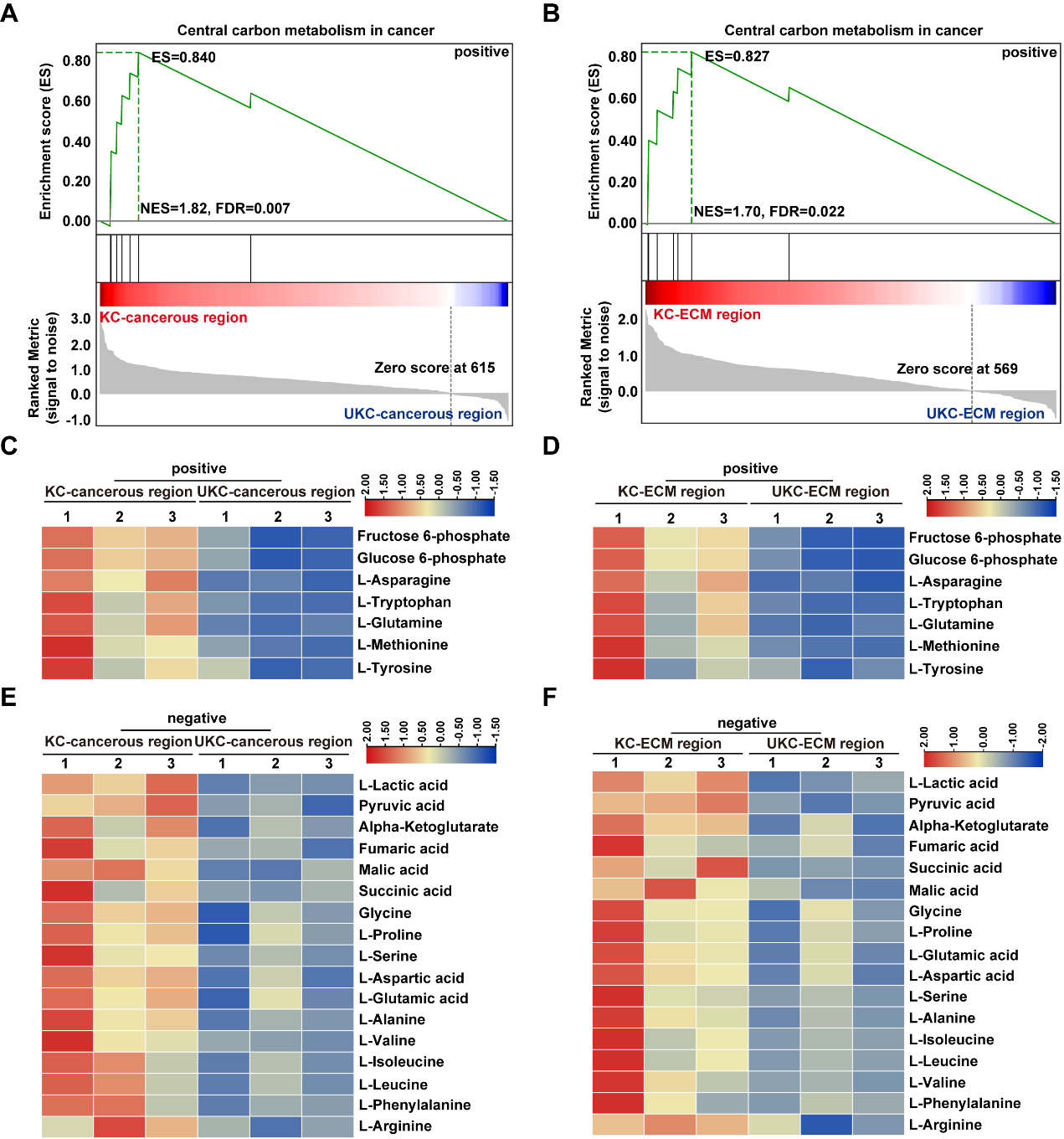


**Figure S5.** UBE2T promotes central carbon metabolism in PDAC. (A, B) GSEA of central carbon metabolism in cancerous (A) and ECM (B) regions with or without *Ube2t* deletion using spatial metabolomics data. (C-F) Differential metabolites of center carbon metabolism in cancerous and ECM regions with or without *Ube2t* deletion detected by spatial metabolomics analysis in positive (C, D) and negative (E, F) condition (*n* = 3).

**
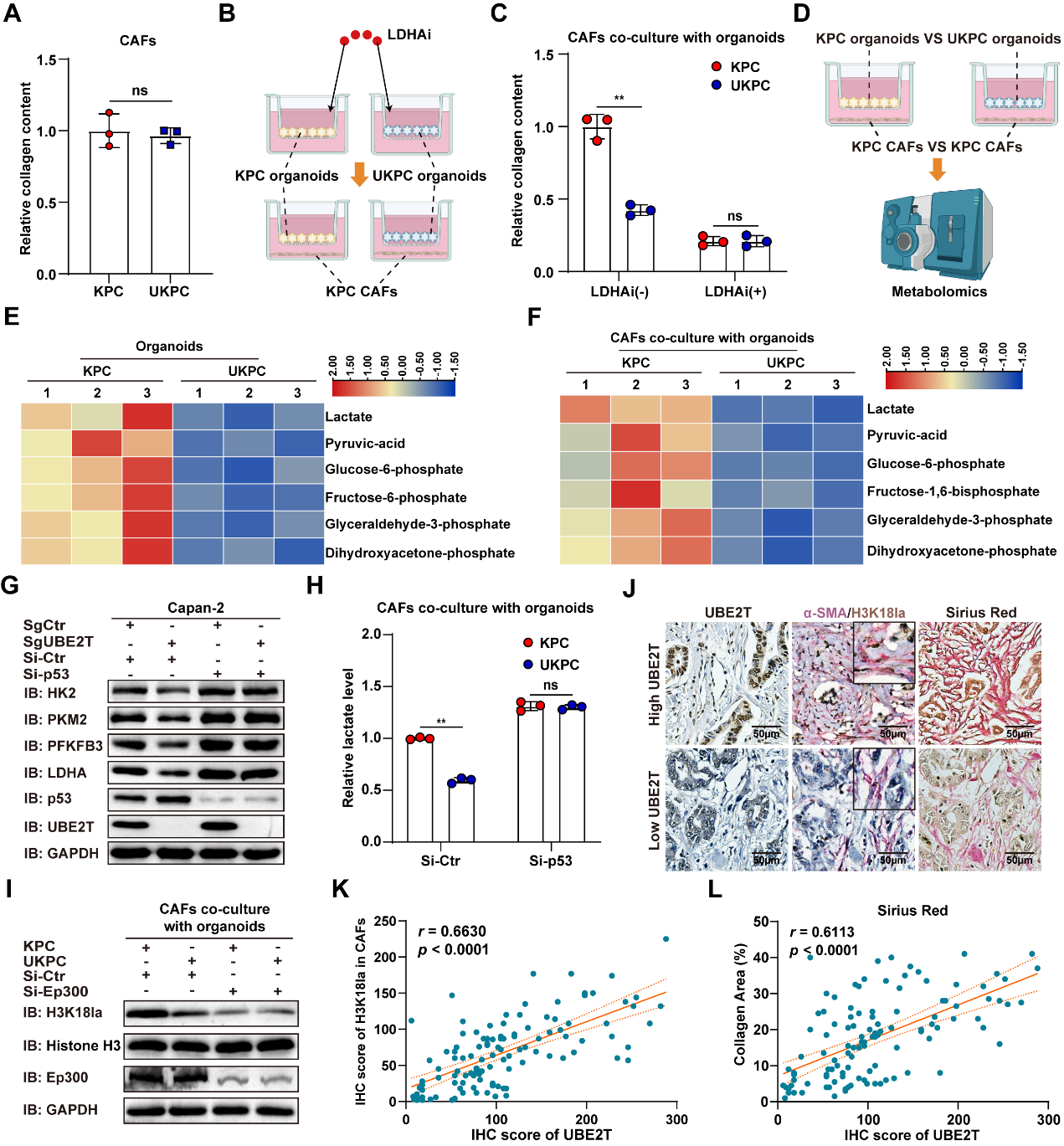
**

**Figure S6.** UBE2T enhances glycolysis to promote CAF H3K18la and stromal deposition in PDAC. (A) Relative collagen content in CAFs with or without *Ube2t* deletion (*n* = 3). (B) Schematic diagram of KPC and UKPC organoids with or without oxamate treatment co-cultured with CAFs. (C) Relative collagen content in CAFs co-cultured with KPC or UKPC organoids treated with or without oxamate (10 mm) (*n* = 3). (D) Schematic diagram of energy metabolomics analysis. (E, F) Differential metabolites of glycolysis in organoids with or without *Ube2t* deletion (E) and co-cultured CAFs (F) detected by energy metabolomics. (G) Protein levels of the indicated proteins in SgCtr and SgUBE2T Capan-2 cells with or without *p53* knockdown. (H) Relative lactate levels of CAFs co-cultured with KPC and UKPC organoids with or without *p53* knockdown (*n* = 3). (I) Protein levels of H3K18la in Si-Ctr and Si-Ep300 CAFs co-cultured with KPC or UKPC organoids. (J-L) Representative images of UBE2T, H3K18la/α-SMA, and Sirius Red staining (J), and correlation analysis between UBE2T with H3K18la/α-SMA (K) or collagen area (L) in PDAC clinical samples. Student's *t* test in A, C, and H, results are presented as the mean ± SD. ^**^*p* < 0.01; ns, no significance.


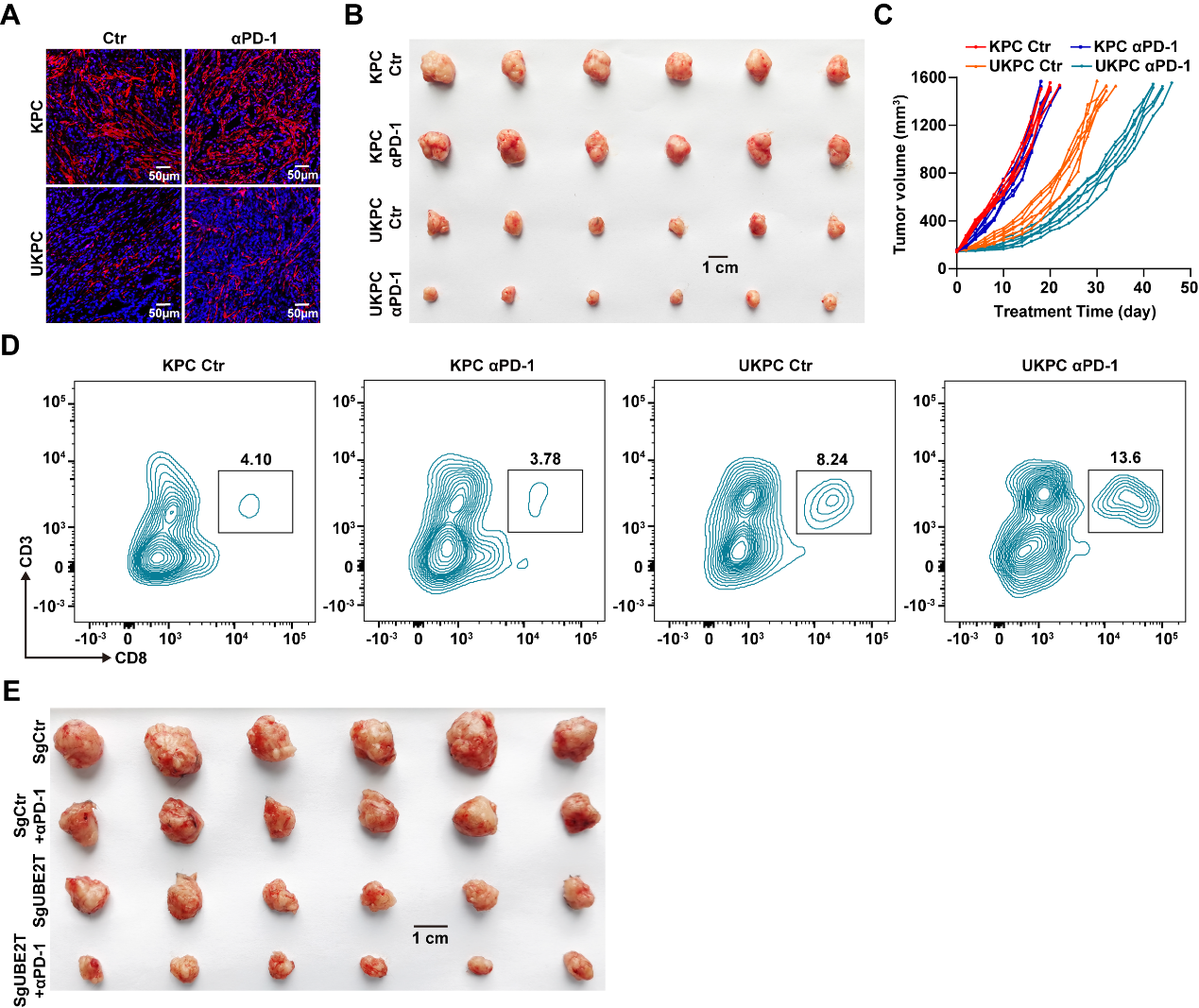


**Figure S7.** *Ube2t* deletion improves anti-PD-1 immunotherapy in PDAC. (A) Representative images of α-SMA staining in KPC and UKPC allografts treated with or without anti-PD-1 therapy. (B) Representative images of tumors in KPC and UKPC allografts treated with or without anti-PD-1 therapy. (C) Tumor growth of KPC and UKPC allografts treated with or without anti-PD-1 therapy (*n* = 6). (D) Representative FCA images of CD3^+^CD8^+^ T cells among CD45^+^ cells in KPC and UKPC allografts treated with or without anti-PD-1 therapy. (E) Representative images of tumors in indicated genotypic Panc02 cells-derived allografts treated with or without anti-PD-1 therapy.


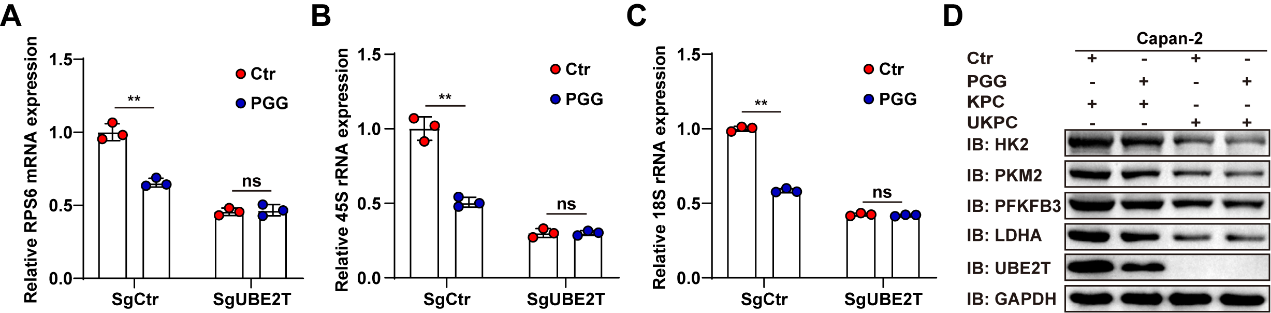


**Figure S8.** PGG inhibits Ribosome biogenesis and glycolysis. (A-C) RPS6 mRNA (A), 45S (B) and 18S rRNA (C) expression levels in SgCtr and SgUBE2T Capan-2 cells with or without PGG (10 µm) treatment (*n* = 3). (D) Protein levels of the indicated proteins in SgCtr and SgUBE2T Capan-2 cells treated with or without PGG. Student's *t* test in A-C, results are presented as the mean ± SD. ^**^*p*< 0.01; ns, no significance.


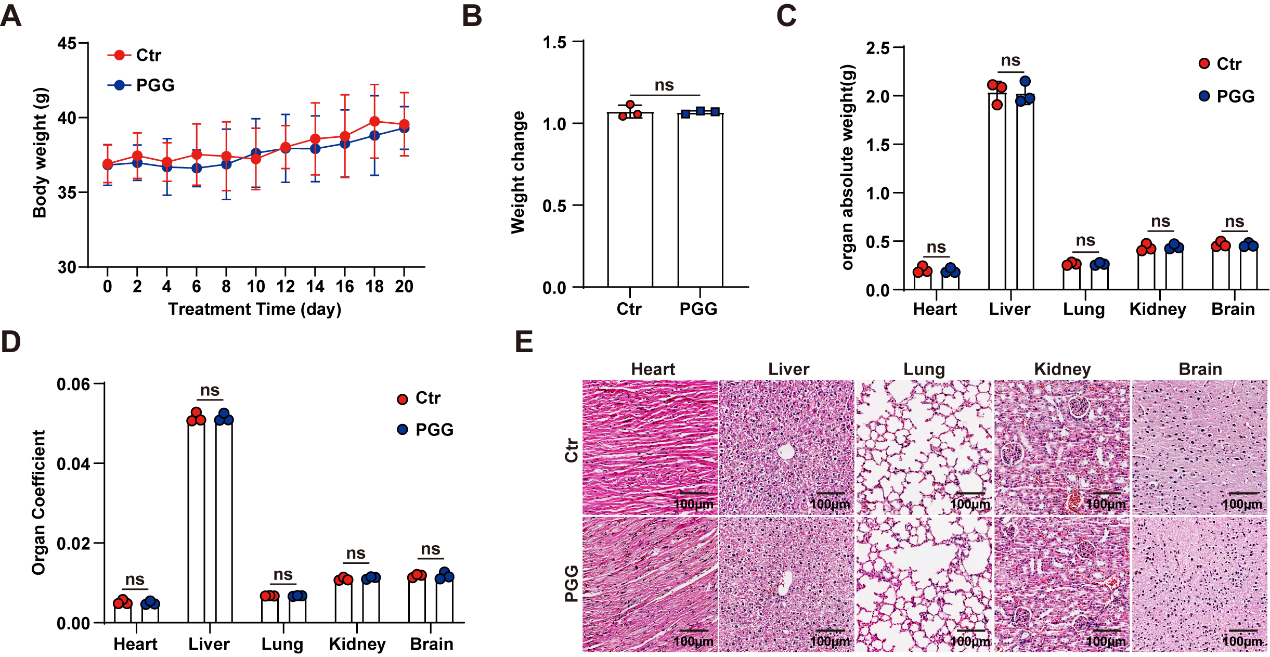


**Figure S9.** Toxicity evaluation of PGG treatment. (A, B) Body weight change of KM mice treated with or without PGG (*n* = 3). (C, D) Organ absolute weight and organ coefficient of KM mice treated with or without PGG (*n* = 3). (E) Representative H&E images of the indicated visceral organs in KM mice treated with or without PGG. Student's *t* test in B-D, results are presented as the mean ± SD. ns, no significance.


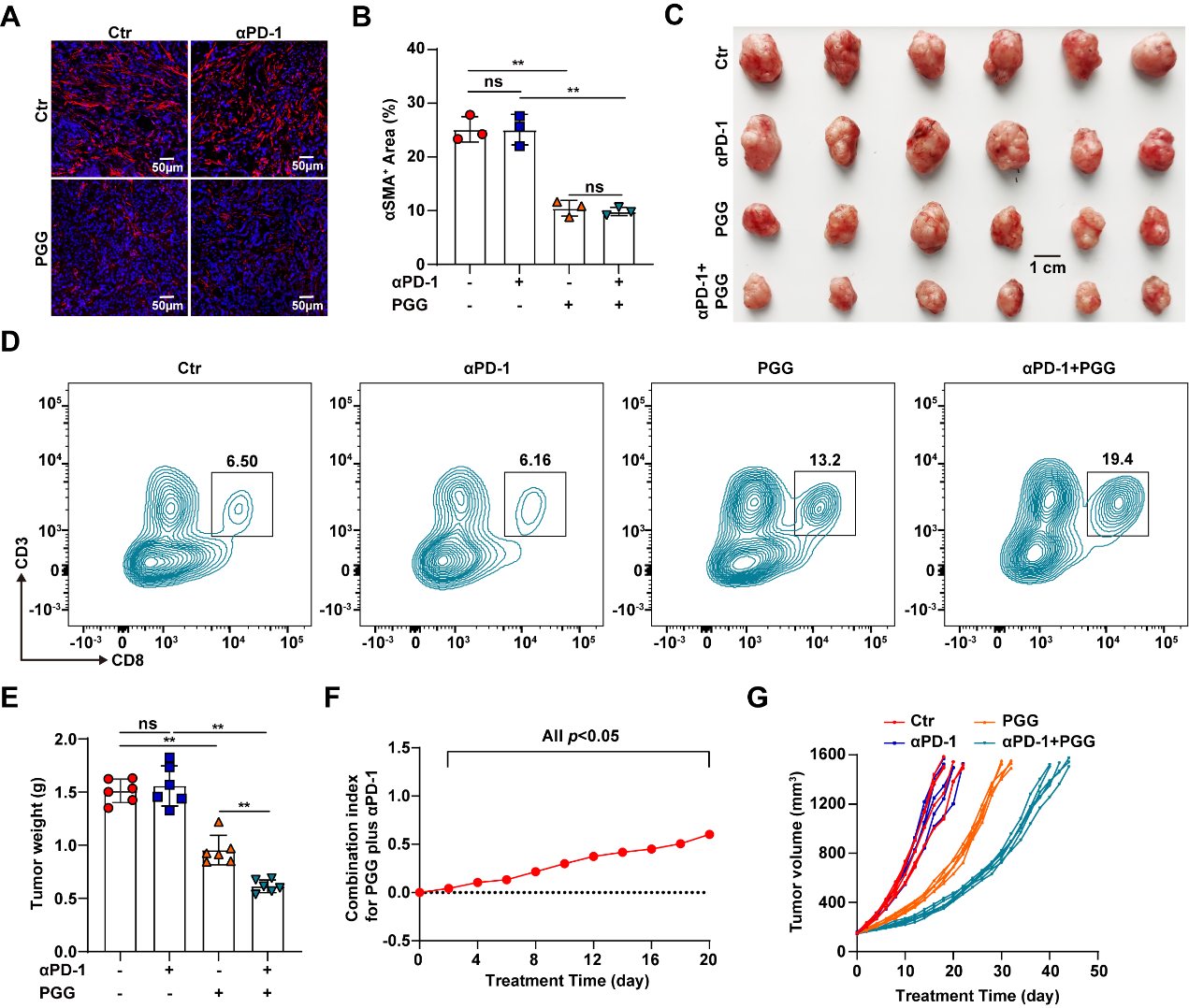


**Figure S10.** PGG synergizes with anti-PD-1 therapy in PDAC. (A, B) Representative images (A) of α-SMA staining and quantification (B) in KPC allografts with or without PGG and/or anti-PD-1therapy (*n* = 3). (C) Representative images of tumors in KPC allografts with or without PGG and/or anti-PD-1therapy. (D) Representative FCA images of CD3^+^CD8^+^ T cells among CD45^+^ cells in KPC allografts with or without PGG and/or anti-PD-1 therapy. (E) Quantification of tumor weight in KPC allografts with or without PGG and/or anti-PD-1therapy (*n* = 6). (F) Drug synergy between PGG and anti-PD-1 antibody was evaluated in KPC allografts using CombPDX. A combination index larger than zero was defined as synergistic. (G) Tumor growth of KPC allografts treated with or without PGG and/or anti-PD-1 therapy (*n* = 6). One-way ANOVA with Bonferroni correction in B and E, results are presented as the mean ± SD. ^**^*p* < 0.01; ns, no significance.


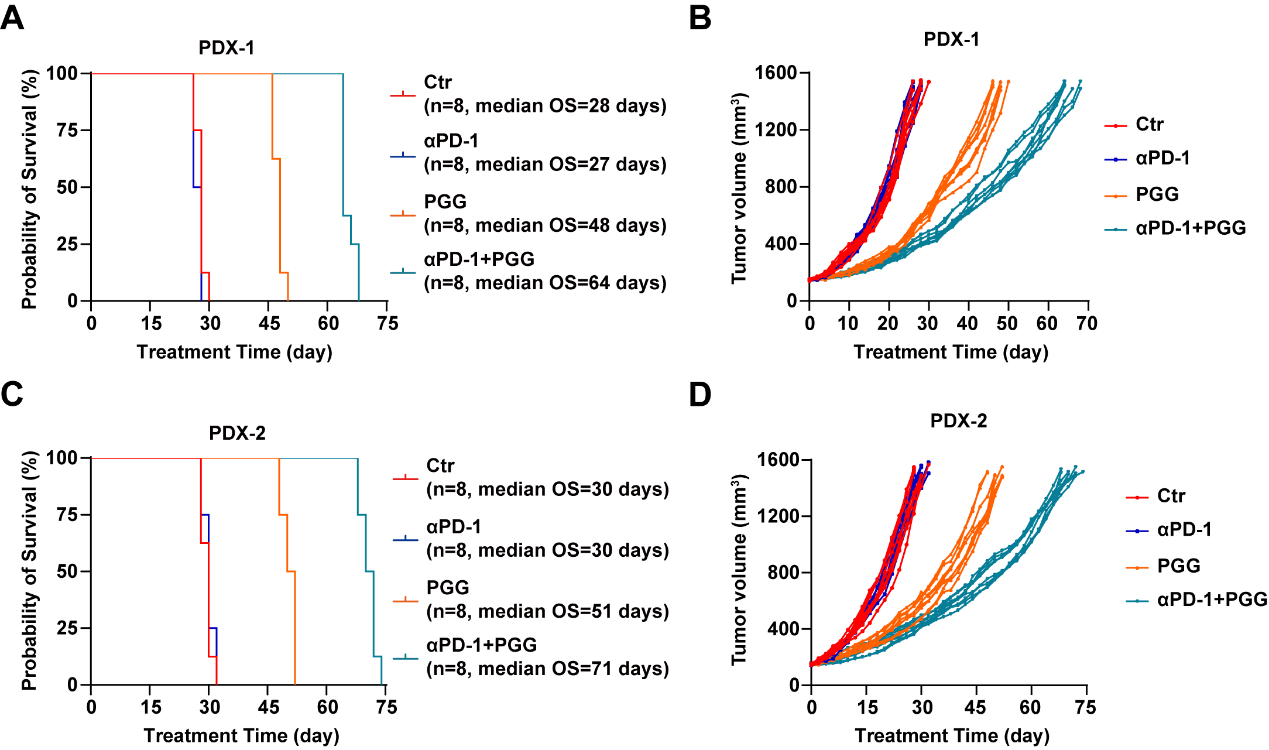


**Figure S11.** The combination of PGG and anti-PD-1 therapy prolongs overall survival in PDXs. (A-D) OS analysis (A, C) and tumor growth (B, D) of PDX-1 and PDX-2 treated with or without PGG and/or anti-PD-1 therapy (*n* = 8).

**Supplemental methods**

*Cell lines*: Capan-2 cells (RRID: CVCL_0026) were purchased from Guangzhou Saiku Biotechnology Co., Ltd (Guangzhou, China). Panc02 cells (RRID: CVCL_D627) were purchased from Wuhan Pricella Biotechnology Co., Ltd. (Wuhan, China). HEK-293T cells (RRID: CVCL_0063) were purchased from the Cell Bank of the Chinese Academy of Sciences (Shanghai, China). Mouse cancer-associated fibroblasts (CAFs) were derived from pancreatic tumor tissue of KPC mice. Briefly, the tumor tissue was minced and digested with Trypsin-EDTA (0.25%) (#25200072, Gibco, New York, USA) for 20-30 minutes, then filtered through a 70 μm cell strainer (#352350, Corning, New York, USA). After centrifugation, the obtained cells were seeded into cell culture dishes. Capan-2 and HEK-293T were grown in Dulbecco’s modified Eagle medium (DMEM) (#C11995500BT, Gibco, New York, USA) supplemented with 10% fetal bovine serum (FBS) (#CF-01S, Cell-Box, Changsha, China). Panc02 and KPC CAFs were cultured in Roswell Park Memorial Institute (RPMI) 1640 medium (#31870082, Gibco, New York, USA) containing 10% FBS. All cell lines were tested regularly to ensure they are mycoplasma-free and validated by short tandem repeat DNA fingerprinting analysis.

*Immunoblotting*: The details of the procedure are described in our previous study [1]. Specific proteins are detected using primary antibodies anti-Flag (#F1804, 1:1000, Sigma-Aldrich, Missouri, USA, RRID: AB_262044), anti-HA (#71-5500, 1:1000, Invitrogen, California, USA, RRID: AB_2533988), anti-His (#SAB1305538, 1:1000, Sigma-Aldrich, Missouri, USA, RRID: AB_2687993), anti-GAPDH (#10494-1-AP, 1:1000, Proteintech, Wuhan, China, RRID: AB_2263076), anti-UBE2T (#10105-2-AP, 1:1000, Proteintech, Wuhan, China, RRID: AB_2211478), anti-RING1 (#13069, 1:1000, CST, Massachusetts, USA, RRID: AB_2713962), anti-MDM2 (#ab259265, 1:1000, Abcam, Cambridge, UK, RRID: AB_2920616), anti-RPL5 (#29092-1-AP, 1:1000, Proteintech, Wuhan, China, RRID: AB_2881240), anti-p53 (#48818S, 1:1000, CST, Massachusetts, USA, RRID: AB_2713958) anti-RPS6 (#2217T, 1:1000, CST, Massachusetts, USA, RRID: AB_331355), anti-L-Lactyl Lysine (#PTM-1401RM, 1:1000, PTM BIO, Hangzhou, China, RRID: AB_2942013), anti-L-Lactyl-Histone H3 (Lys18) (#PTM-1427RM, 1:1000, PTM BIO, Hangzhou, China, RRID: AB_3076698), anti-Histone H3 (#PTM-1001RM, 1:1000, PTM BIO, Hangzhou, China, RRID: AB_3676032), anti-Histone H4 (#PTM-1015RM, 1:1000, PTM BIO, Hangzhou, China, RRID: AB_3101866), anti-L-Lactyl-HistoneH3 (Lys9) (#PTM-1419RM, 1:1000, PTM BIO, Hangzhou, China, RRID: AB_3076695), anti-L-Lactyl-HistoneH3(Lys23) (#PTM-1413RM, 1:1000, PTM BIO, Hangzhou, China, RRID: AB_3101865), anti-L-Lactyl-HistoneH4 (Lys5) (#PTM-1407RM, 1:1000, PTM BIO, Hangzhou, China, RRID: AB_3096309), anti-L-Lactyl-HistoneH4 (Lys8) (#PTM-1415RM, 1:1000, PTM BIO, Hangzhou, China, RRID: AB_3101829), anti-L-Lactyl-HistoneH4 (Lys12) (#PTM-1411RM, 1:1000, PTM BIO, Hangzhou, China, RRID: AB_2941896), anti-p300 (#PTM-20295, 1:1000, PTM BIO, Hangzhou, China), anti-DPP4 (#85159-3-RR, 1:1000, Proteintech, Wuhan, China), anti-LDHA (#19987-1-AP, 1:1000, Proteintech, Wuhan, China, RRID: AB_10646429), anti-HK2 (#22029-1-AP, 1:1000, Proteintech, Wuhan, China, RRID: AB_11182717), anti-PFKFB3 (#13763-1-AP, 1:1000, Proteintech, Wuhan, China, RRID: AB_2162854) and anti-PKM2 (#60268-1-lg, 1:1000, Proteintech, Wuhan, China).

*Cycloheximide (CHX) assay*: Capan-2 cells of specified genotypes were treated with 100 μg mL^-1^ CHX (#HY-12320, MCE, New Jerse, USA). Total protein was extracted from untreated cells and cells treated with CHX for 2, 4, 6, and 8 hours. Western blotting was used to detect the expression level of p53.

*Co-immunoprecipitation*: pCMV3-Flag-MDM2 and pCMV3-His-RPL5 were transfected into Capan-2 cells with specific genotypes or drug treatments, and the subsequent procedures were performed as previously described [1].

*In vivo ubiquitination assay*: pCMV-Flag-p53, pCMV-Flag-RPS6, pCMV-HA-ubiquitin (WT), pCMV-HA-ubiquitin (K48R), pCMV-notag-RING1, pCMV-notag-UBE2T, pCMV3-notag-MDM2 and pCMV3-His-RPL5 were transfected into Capan-2 cells or HEK-293T cells with specific treatments, and the subsequent process is described in our previous study [1].

*Quantitative Real-Time Polymerase Chain Reaction (qRT-PCR)*: EasyPure® Fast Cell RNA Kit (#ER111-02, TransGen Biotech, Beijing, China) was used to extract total RNA from cells. PrimeScript™ RT reagent Kit (Perfect Real Time) (#RR037A, Takara Bio, Tokyo, Japan) was used to generate cDNA. qRT-PCR was performed using the TB Green^®^ Premix Ex Taq™ (Tli RNaseH Plus) (#RR420Q, Takara Bio, Tokyo, Japan) in a Rotor-Gene Q real-time PCR cycler (#9001862, QIAGEN, Duesseldorf, Germany). GAPDH mRNA levels were employed as controls. The relative expression levels were calculated using the 2^−ΔΔCt^ method.

*Plasmids and Small interfering RNA*: pCMV3-notag-MDM2 (#HG11206-UT), pCMV3-Flag-MDM2 (#HG11206-CF), pCMV3-His-RPL5 (#HG16280-CH) and pCMV3-Flag-RPS6 (#HG18196-CF) were purchased from Sino Biological Inc. (Beijing, China). pCMV-HA-ubiquitin (WT), pCMV-HA-ubiquitin (K48R), pCMV-notag-RING1, pCMV-notag-UBE2T and pCMV-Flag-p53 plasmids were constructed in our previous study [2]. Transfection of these plasmids was carried out using Lipofectamine™ 2000 Transfection Reagent (#11668019, Invitrogen, California, USA) following the manufacturer’s protocol. Small interfering RNA for *Ep300* (#siB12329161853-1-5), *Ldha* (#siG150519174059-1-5), *RPL5* (#stB0007722A-1-5), *MDM2* (#stB0001232A-1-5), *TP53* (#stB0002017A-1-5), *Trp53* (#siB151230043809-1-5) and corresponding controls (#siT0000001-1-5) were purchased from RiboBio (Guangzhou, China) and transfected according to the manufacturer’s instructions.

*Lentiviral Infection*: Lentivirus of *UBE2T* knockout were purchased from Shanghai Genechem Co., Ltd (Shanghai, China). The sequences of the guide RNAs (SgRNAs) of *UBE2T* and the knockout efficiency have been described in our previous study [2].

*Collagen detection*: The organoids and fibroblasts were co-cultured in 0.4 μm transwell systems. The basement membrane matrix hydrogel (#BME001, Bio-techne, Minnesota, USA) was spread evenly on the microporous membrane and was left overnight to solidify. Organoids of specific genotypes were seeded into the upper chambers and cultured under indicated treatments for 24 hours. Meanwhile, CAFs of specific genotypes were seeded in the lower chambers of other transwell compartments and cultured separately until the cell density reached 70-80%. The CAFs and organoids were then co-cultured for 24 hours after the medium was renewed. After that, the collagen content in the culture medium of CAFs was quantified using the Sirius Red Total Collagen Detection Kit (#9062, Chondrex, Washington, USA) according to the official protocol.

*Lactate detection*: Organoids of specific genotypes were cultured under indicated treatments in the upper chambers of transwell compartments for 24 hours, and CAFs of specific genotypes were cultured in lower chambers of other compartments simultaneously until the cell density reached 70-80%. After that the cultural medium was renewed, and the CAFs and organoids were co-cultured for 24 hours. Next, medium lactate assay was performed after the organoids and the CAFs were cultured separately in new medium for 24 hours. The lactate content in the culture medium was quantified using the Lactate-Glo-Assay Kit (#J5021, Promega, Wisconsin, USA) according to the technical manual.

*Flow cytometry*: Fresh tumor tissue was minced adequately and digested in a buffer containing 1 mg mL^-1^ Collagenase D (#11088866001, Sigma-Aldrich, Missouri, USA) and 0.1 mg mL^-1^ DNase I (#LS002006, Worthington, New Jerse, USA) in DMEM (#C11995500BT, Gibco, New York, USA) for 40-60 min at 37°C with continuous shaking. After incubation, collagenase was inactivated by adding an equal volume of DMEM supplemented with 10% FBS. After centrifugation the PBS buffer was discarded, and the cell pellets were resuspended in Red Blood Cell Lysis Buffer (#NH4CL2009, TBDscience, Tianjin, China) and incubated on ice for 5 min. After centrifugation again, cells were incubated with FC blocking reagent containing purified anti‐mouse CD16/CD32 antibody (#14‐0161‐86, Invitrogen, California, USA, RRID: AB_467133) at 20°C for 10-20 min. Subsequently, the cells were incubated with a mixture of 7‐AAD Viability Staining Solution (#00‐6993‐50, Invitrogen, California, USA) and fluorophore‐conjugated antibodies including APC/Cyanine7 anti-mouse CD45 (#103116, 1:50, Biolegend, California, USA, RRID: AB_312981), FITC anti-mouse CD3 (#100204, 1:50, Biolegend, California, USA, RRID: AB_312661) and APC anti-mouse CD8α Antibody (#100712, 1:50, Biolegend, California, USA, RRID: AB_312751) for 30 minutes at room temperature in the dark to mark CD8 positive T cells. After centrifugation, the cells were resuspended in PBS buffer and analyzed using a BD FACS Canto Flow Cytometer (BD Biosciences, USA). All flow cytometry data were analyzed using FlowJo software (BD Life Sciences, USA).

*Immunohistochemistry*: Paraffin sections were deparaffinized in xylene and hydrated in graded ethanol solution, followed by antigen retrieval using citrate buffer (pH 6.0). The subsequent processes were performed using the UltraSensitive™ SP (mouse/rabbit) IHC kit (#KIT-9710, MXB, Fuzhou, China) according to the manufacturer’s protocol, followed by color reaction using diaminobenzidine (DAB) (#DAB-0031, MXB, Fuzhou, China) and counterstaining with hematoxylin. Immunohistochemical double staining was conducted using the Double Stain Kit (ZSGB-BIO, Cat#DS-0003) in accordance with the manufacturer’s protocol. The primary antibodies include anti-CD8α (#98941, 1:100, CST, Massachusetts, USA, RRID: AB_2756376), anti-L-Lactyl-Histone H3 (Lys18) (#PTM-1427RM, 1:100, PTM BIO, Hangzhou, China, RRID: AB_3076698), anti-α-SMA (#A2547, 1:200, Sigma-Aldrich, Missouri, USA, RRID: AB_476701) and anti-UBE2T (#NBP2-02965, 1:100, Novus Biologcials, Colorado, USA, RRID: AB_3076689). The staining intensity (0, no staining; 1, weak staining; 2, moderate staining; 3, strong staining) and the percentage of positive area (0-100%) were assessed by two pathologists independently.

*Immunofluorescence*: Paraffin sections were deparaffinized and hydrated, followed by antigen retrieval using citrate buffer (pH 6.0). Next, the sections were immersed in QuickBlock™ Blocking Buffer for Immunol Stainin (#P0260, Beyotime, Shanghai, China) at room temperature for 1 hour. Subsequently, the sections were incubated with primary antibodies at 4°C overnight. The next day, the sections were incubated with secondary antibodies at 37°C for 1 hour after washed with PBS buffer. TISSUE FAXS PLUS (TissueGnostics, Austria) was used to capture the images of histopathologic slides. The cell nucleus is stained with DAPI (#C0060, 1:500, Solarbio, Beijing, China). The antibodies used include anti-α-SMA (#A2547, 1:200, Sigma-Aldrich, Missouri, USA, RRID: AB_476701), goat anti-rabbit IgG H&L (Alexa Fluor® 488) (#ab150077, 1:200, Abcam, Cambridge, UK, RRID: AB_2630356) and goat anti-mouse IgG H&L (Alexa Fluor® 594) (#ab150116, 1:200, Abcam, Cambridge, UK, RRID: AB_2650601).

*Sirius red staining*: After deparaffinisation and hydration, the slides were stained using Modified Sirius Red Stain Kit (No Picric Acid) (#G1472, Solarbio, Beijing, China) according to the manufacturer’s protocol. The percentage of Sirius red-positive areas was measured using ImageJ.

*In vivo toxicity assessment of PGG*: Eight-week-old female KM mice were purchased from the Lanzhou Veterinary Research Institute, Chinese Academy of Agricultural Sciences, and randomly divided into two groups. The treatment group was orally administered PGG (#HY-N0527, MCE, New Jersey, USA) at a dosage of 20 mg kg^-1^ every day, while the control group received an equal volume of vehicle. Body weights were recorded every two days throughout the 20-day experimental period. At the end of the experiment, mice were euthanized, and organs were harvested, weighed, and fixed for histopathological examination. Tissue sections were stained with H&E to assess potential organ damage.

*Transcriptomic analysis*: Total RNA of KPC CAFs (*n* = 3) with specific treatment was extracted using Trizol method and was identified and quantified using a Qubit fluorescence quantifier (Thermo Fisher Scientific, USA) and a Qsep 400 high-throughput biofragment analyzer (BiOptic Inc., China). Oligo (dT) magnetic beads were used to enrich mRNAs, and the cDNA was synthesized via reverse transcription PCR using fragmented mRNA and a random hexamer primer, followed by library construction through end repair, sequencing adapter ligation, fragment selection, PCR amplification and purification. Circular DNA templates were synthesized from cDNA by single-stranded cyclization. Then DNA Nano Balls (DNBs) were generated via amplification using phi 29 polymerase and were loaded onto a chip for high-throughput sequencing. Fastp was used to filter low-quality reads (with adapters, N > 10% or low-quality bases (Q ≤ 20) > 50%). Clean reads were aligned to the reference genome using HISAT. Gene expression levels were quantified using featureCounts and subsequently the FPKM (Fragments Per Kilobase Million) values for each gene were computed based on gene length. Differential gene expression analysis between the two groups was performed using DESeq2 with *p*-values corrected using the Benjamini & Hochberg method, and GO/KEGG enrichment analysis was conducted based on the hypergeometric test.

*Cleavage Under Targets and Tagmentation (CUT&Tag)*: CUT&Tag assay was performed by OE Biotech Co., Ltd. (Shanghai, China). CUT&Tag library was constructed using Hyperactive^TM^ In-Situ ChIP Library Prep Kit for Illumina (#TD903-TD904, Vazyme Biotech, Nanjing, China). Briefly, cells were bound with Concanavalin A-coated magnetic beads (ConA beads), and Digitonin was used to penetrate the cell membrane. Cells were then incubated with anti-H3K18la (PTM-1427RM, PTM Bio, Hangzhou, China, RRID: AB_3076698), the secondary antibody, and Protein A/Protein G fused with the Tn5 transposon (Hyperactive pA/pG-Tn5 Transposon) that ligates P5 and P7 adaptors to the target DNA fragments. The sequencing library was constructed by PCR amplification using P5 and P7 primers. After that, the purified PCR products were qualified by the Agilent 2100 Bioanalyzer (Agilent Technologies, Santa Clara, CA, USA) and sequenced using the Illumina NovaSeq6000 platform (Illumina, Inc., America), generating 150 bp paired-end reads for subsequent analysis. Fatsp was used to obtain the clean reads that were subsequently aligned to the reference genome using Bowtie 2. The SEACR software was used to detect genomic regions enriched for multiple overlapping DNA fragments (peaks) based on the “stringent” parameter, and Chipseeker was used to annotate the peaks. Differential analysis was performed using Manorm.

*Energy metabolomics*: All metobolites were detected by Metware Biotechnology Inc (Wuhan, China), based on the QTRAP® 6500 LC-MS/MS System (AB Sciex Pte. Ltd., Singapore). Briefly, the samples of organoids cultured under specified treatments (n=3) and co-cultured CAFs (n=3) were thawed on ice and total metabolites were extracted using pre-cooled methanol (-20°C) through three freeze-thaw cycles (liquid nitrogen/ice) followed by 20 minutes of standing at -20°C. After centrifugation (12,000 rpm, 10 min, 4°C), the supernatant was purified using a protein precipitation plate for LC-MS analysis. Liquid Chromatography was achieved on a ACQUITY UPLC BEH Amide column (Waters Corporation, America) with a mobile phase consisting of (A) water containing 10 mm ammonium acetate and 0.3% ammonium hydroxide and (B) 90% acetonitrile/water (V/V). The gradient elution was started at 95% B (0-1.2 min), decreased to 70% B (8 min), 50% B (9-11 min), finally ramped back to 95% B (11.1-15 min), with a flow rate of 0.4 mL min^-1^ at 40°C. Mass spectrometry was conducted on a QTRAP® 6500+ system (AB Sciex Pte. Ltd., Singapore). The Electrospray Ionization (ESI) temperature was set to 550°C and the ion spray voltage is 5500 V in the positive mode and -4500 V in the negative mode. The metabolites were qualified based on Metware Database and quantified via Multiple Reaction Monitoring (MRM) mode of Triple Quadrupole Mass Spectrometer (QqQ-MS). Data analysis included Principal Component Analysis (PCA) (prcomp package), Partial Least Squares-Discriminant Analysis (PLS-DA) (MetaboAnalystR package) and KEGG (RRID: SCR_012773) pathway annotation with enrichment significance assessed by the hypergeometric test. The Benjamini–Hochberg procedure to control the false discovery rate (FDR).

*Spatial metabolomics analysis*: Pancreatic tissues from 8-month-old KC (n = 3) and UKC (n = 3) mice were removed surgically and embedded with tissue freezing medium (Leica Microsystem, Germany) for spatially resolved metabolomic analysis and bioinformatics analysis by Lumingbio (Shanghai, China). The detailed process is as described previously [3]. Briefly, after storage at -80°C, samples were employed to prepare 10 μm serial sections using a cryostat microtome (Leica CM 1950, Leica Microsystems, Germany), and the sections were thaw-mounted on positive charge desorption plate (Thermo Scientific, U.S.A) and stored at -80°C. The sections were desiccated at -20°C and then at room temperature before detection, and then scanned and analyzed with an AFADESI-MSI platform (Beijing Victor Technology Co., LTD, Beijing, China) in tandem with a Q-Orbitrap mass spectrometer (Q Exactive, Thermo Scientific, U.S.A.). The solvent formulation was acetonitrile (ACN)/H2O (8:2) in the negative mode and ACN/H2O (8:2, 0.1% FA) in the positive mode, with a spray voltage of 7 kV, a capillary temperature of 350°C, a scanning range of 70-1000 Da, and a spatial resolution of 50 μm (X-axis scanning speed of 0.2 mm s^-1^). The mass spectrometry data were converted to “. imzML” format and then subjected to total ion count normalization (TIC) using MSiReader and background subtraction using the Cardinal 3 software package. Region-specific MS profiles were accurately extracted by matching high spatial resolution Sirius red-stained images. Differential metabolite screening was accomplished by orthogonal partial least squares discrimination analysis (OPLS-DA) (VIP>1) combined with t-test (P<0.05), and metabolic characteristics of tissue microregions were resolved using T-distributed stochastic neighbor embedding (t-SNE) combined with uniform manifold approximation and projection for dimension reduction (UMAP) downscaling and SSCC spatial clustering algorithms. Metabolites identification was achieved by matching the SmetDB local database with the pySM spatial metabolome annotation framework. In the GSEA analysis based on differential metabolites, the Benjamini-Hochberg procedure was used to control the false discovery rate (FDR).

*Single-cell RNA sequencing analysis*: Control and oxamate-treated KPC allografts (n=3) were subjected to single-cell RNA sequencing. The FASTQ files were processed and aligned to GRCm39 mice reference genome using Cell Ranger software (version 9.0.1) from 10x Genomics, with unique molecular identifier (UMI) counts summarized for each barcode. The UMI count matrix was then analyzed using Seurat (version 4.0.0) R package. To remove low-quality cells and likely multiplet captures, cells were filtered by (1) gene numbers < 200, (2) UMI <1000, (3) log10GenesPerUMI < 0.7, (4) proportion of UMIs mapped to mitochondrial genes > 10% and (5) percentage of proportion of UMIs mapped to hemoglobin genes > 5%. Subsequently, the DoubletFinder package (version 2.0.3) was used to identify potential doublets, and library size normalization was processed using the NormalizeData function to obtain the normalized gene expression data. Top2000 highly variable genes were selected using the FindVariableGenes function, and principal component analysis was conducted using the expression profiles of these highly variable genes with RunPCA function. Then, the RunHarmony function in harmony (version 1.0) R package was performed to remove the batch effects. Graph-based clustering was performed to cluster cells according to their gene expression profile with the FindClusters function. Single-cell cluster visualization was performed using a 2-dimensional Uniform Manifold Approximation and Projection (UMAP) algorithm with the RunUMAP function. Marker gene identification was carried out with the FindAllMarkers function. The singleR package (version 1.4.1) was used to calculate the correlation between the expression spectrum of the cells to be identified and the reference data set, and then the cell types with the highest correlation in the reference data set were assigned to the identified cells. Differentially expressed genes (DEGs) were identified using the FindMarkers function. A threshold of *P* value < 0.05 and |log2FC| > 0.58 was set for significantly differential expression. Functional analysis of CAF subtypes was conducted based on the top 100 marker genes. Negative regulation of immune response for CAF subtypes was evaluated using the GO:0050777 dataset. The immunosuppressive activity of Treg cells was assessed using a gene set comprising FOXP3, CTLA4, LAG3, PD-1, TIGIT, IKZF2, TNFRSF4, TNFRSF18, ICOS, CD25, TGF-β, IL10, IL35, CD39, and CD73.Cell trajectory analysis was performed using the Monocle package (version 2.9.0). Cell-cell communication between CAFs subsets and T-NK cells subsets was inferred and analyzed using the CellChat (version 1.6.1). Sequencing and bioinformatics analysis were provided by OE Biotech Co., Ltd. (Shanghai, China).

**References**

[1] Z. Yu, X. Jiang, L. Qin, et al., *"*A Novel UBE2T Inhibitor Suppresses Wnt/β‐catenin Signaling Hyperactivation and Gastric Cancer Progression by Blocking RACK1 Ubiquitination,*"* *Oncogene* 40, no. 5 (2021): 1027-1042.

[2] X. Jiang, Y. Ma, T. Wang, et al., *"*Targeting UBE2T Potentiates Gemcitabine Efficacy in Pancreatic Cancer by Regulating Pyrimidine Metabolism and Replication Stress,*"* *Gastroenterology* 164, no. 7 (2023): 1232-1247.

[3] X. Jiang, T. Wang, B. Zhao, et al., *"*KRASG12D-Driven Pentose Phosphate Pathway Remodeling Imparts a Targetable Vulnerability Synergizing with MRTX1133 for Durable Remissions in PDAC,*"* *Cell Reports Medicine 6*, no. 2 (2025): 101966.
